# Patterns of CRISPR/Cas9 activity in plants, animals and microbes

**DOI:** 10.1111/pbi.12634

**Published:** 2016-10-11

**Authors:** Luisa Bortesi, Changfu Zhu, Julia Zischewski, Lucia Perez, Ludovic Bassié, Riad Nadi, Giobbe Forni, Sarah Boyd Lade, Erika Soto, Xin Jin, Vicente Medina, Gemma Villorbina, Pilar Muñoz, Gemma Farré, Rainer Fischer, Richard M. Twyman, Teresa Capell, Paul Christou, Stefan Schillberg

**Affiliations:** ^1^ Institute for Molecular Biotechnology RWTH Aachen University Aachen Germany; ^2^ Department of Plant Production and Forestry Science School of Agrifood and Forestry Science and Engineering (ETSEA) University of Lleida‐Agrotecnio Center Lleida Spain; ^3^ Fraunhofer Institute for Molecular Biology and Applied Ecology IME Aachen Germany; ^4^ TRM Ltd York UK; ^5^ ICREA Catalan Institute for Research and Advanced Studies Barcelona Spain

**Keywords:** genome editing, mutational signature, off‐target mutations, on‐target mutations, sgRNA design, site‐directed mutagenesis, species‐dependent effects

## Abstract

The CRISPR/Cas9 system and related RNA‐guided endonucleases can introduce double‐strand breaks (DSBs) at specific sites in the genome, allowing the generation of targeted mutations in one or more genes as well as more complex genomic rearrangements. Modifications of the canonical CRISPR/Cas9 system from *Streptococcus pyogenes* and the introduction of related systems from other bacteria have increased the diversity of genomic sites that can be targeted, providing greater control over the resolution of DSBs, the targeting efficiency (frequency of on‐target mutations), the targeting accuracy (likelihood of off‐target mutations) and the type of mutations that are induced. Although much is now known about the principles of CRISPR/Cas9 genome editing, the likelihood of different outcomes is species‐dependent and there have been few comparative studies looking at the basis of such diversity. Here we critically analyse the activity of CRISPR/Cas9 and related systems in different plant species and compare the outcomes in animals and microbes to draw broad conclusions about the design principles required for effective genome editing in different organisms. These principles will be important for the commercial development of crops, farm animals, animal disease models and novel microbial strains using CRISPR/Cas9 and other genome‐editing tools.

## Introduction

Clustered regularly interspaced short palindromic repeats (CRISPRs) are repetitive sequences found in bacterial and archaeal genomes interrupted by spacers captured from previously encountered virus genomes and other invasive DNA. Their function is to provide a form of adaptive immunity via CRISPR‐associated (Cas) proteins that act as RNA‐directed endonucleases to degrade the same type of invasive DNA if it is encountered again (Lee *et al*., 2015). Three major CRISPR/Cas systems have been described (Kumar and Jain, 2015) although several additional systems have been reported more recently (Makarova *et al*., 2011, 2015). In type II systems (Jinek *et al*., 2012), fragments of invasive DNA (protospacers) approximately 20 bp in length are captured due to their proximity to a short and highly degenerate sequence known as a protospacer adjacent motif (PAM) and these fragments become the spacers in the genomic CRISPR array. Transcription of the array yields a long transcript which is processed into shorter CRISPR RNAs (crRNAs), each representing a single spacer. The crRNA forms a complex with endonuclease Cas9 and a transactivating crRNA (tracrRNA) which mediates the interaction. The Cas9 ribonucleoprotein (RNP) complex then binds to DNA containing a PAM and a protospacer matching the crRNA. Cleavage occurs three nucleotides upstream of the PAM on both strands, mediated by the Cas9 endonuclease domains RuvC and HNH, respectively, introducing a precise double‐strand break (DSB) with blunt ends that causes target DNA degradation (Chen and Gao, 2014; Doudna and Charpentier, 2014; Osakabe and Osakabe, 2015).

**Figure 1:**
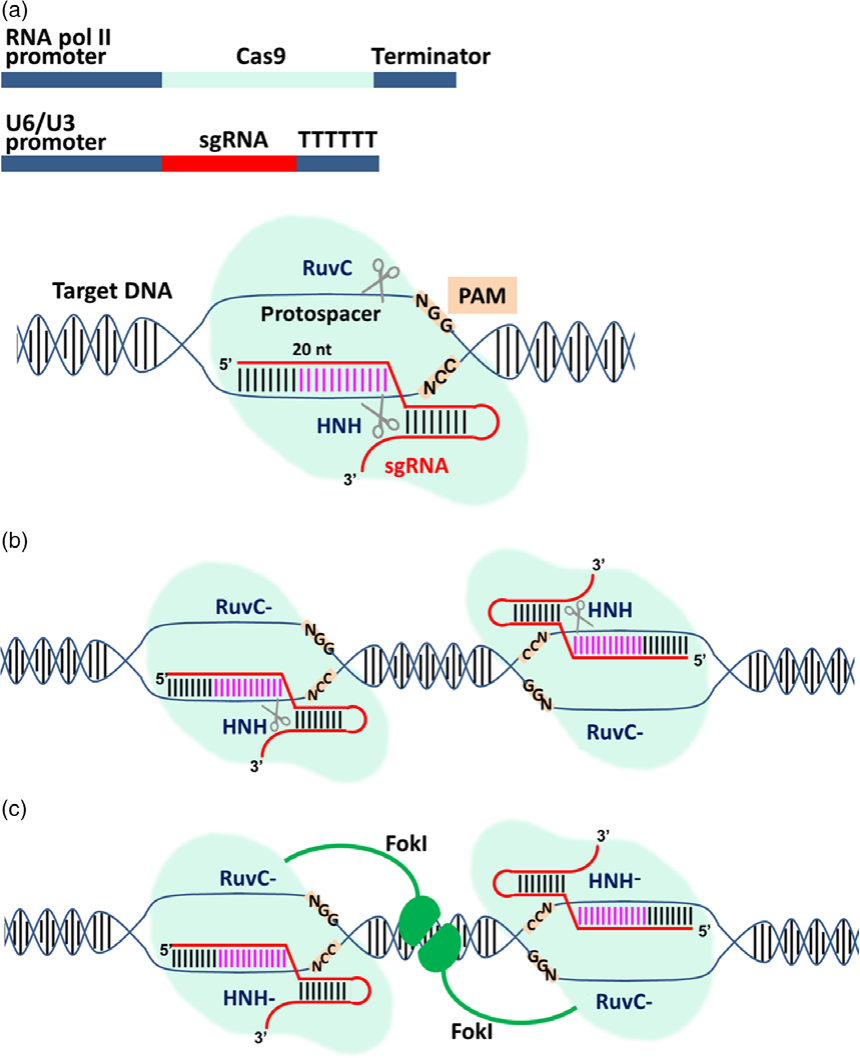
The engineered CRISPR/Cas9 system for genome editing. (a) Outline of the two components required for targeted cleavage: delivery of DNA constructs for transcription of the Cas9 nuclease by RNA polymerase II and the synthetic guide RNA (sgRNA) by RNA polymerase III (usually the U6 or U3 promoter) is the most common procedure, especially in plants. Alternatively, the two components can be provided as RNA or directly as a ribonucleoprotein complex (RNP; not shown). The sgRNA contains a 20‐nt‐long sequence complementary to the genomic target (protospacer). When the Cas9/sgRNA complex finds a matching target in the genome followed by an NGG stretch called protospacer adjacent motif (PAM), the two endonuclease domains in Cas9 (RuvC and HNH) cleave the noncomplementary and complementary strands in the target, respectively, generating a blunt double‐strand break (DSB) 3‐bp upstream of the PAM. The part of sgRNA proximal to the PAM (in pink) is called the seed region, and base pairing with the protospacer in this region is strictly required for recognition and cleavage of the target. Mismatches in the PAM‐distal region are tolerated to some extent. (b) One endonuclease domain can be mutated (e.g. RuvC with the D10A mutation as shown in the figure or HNH with the H840A mutation, not shown) generating a Cas9 nickase. Using two sgRNAs matching adjacent genomic regions, a staggered DSB can be generated by two paired nickases. (c) If both endonuclease domains of Cas9 are mutated, the enzyme becomes catalytically inactive and is called dead Cas9 (dCas9). The dCas9 protein can still bind at its target and if fused to a nonspecific endonuclease such as FokI can generate staggered DSBs. In both (b) and (c), two precisely disposed protospacers have to be found in the genome for cleavage to occur, greatly reducing the number of possible off‐target effects.

The ability of the type II CRISPR/Cas9 system to recognize specific DNA targets has been exploited to develop an RNA‐guided genome‐editing platform that is more versatile than equivalent platforms involving protein‐based DNA‐binding modules such as zinc finger nucleases (ZFNs) and transcription activator‐like effector nucleases (TALENs; Hsu *et al*., 2014). The natural system has been converted into a universal genome‐editing platform by reducing it to two convenient components (Figure 1a). The first is the Cas9 endonuclease, typically from *Streptococcus pyogenes* (*Sp*Cas9), which for eukaryotic targets is equipped with a nuclear localization signal (Belhaj *et al*., 2013, 2015). The second is a synthetic guide RNA (sgRNA) combining the tracrRNA and crRNA functions of the natural system into a single molecule (Belhaj *et al*., 2015; Sander and Joung, 2014). The sgRNA targets a unique 20‐bp sequence in the genome of the host organism, and any sequence can be chosen as long as it is adjacent to a PAM. Codon‐optimized versions of the *cas9* gene offer maximum activity in different host species (Bortesi and Fischer, 2015) although the wild‐type Cas9 is also active in heterologous systems such as rice protoplasts (Jiang *et al*., 2013b).

**Figure 2:**
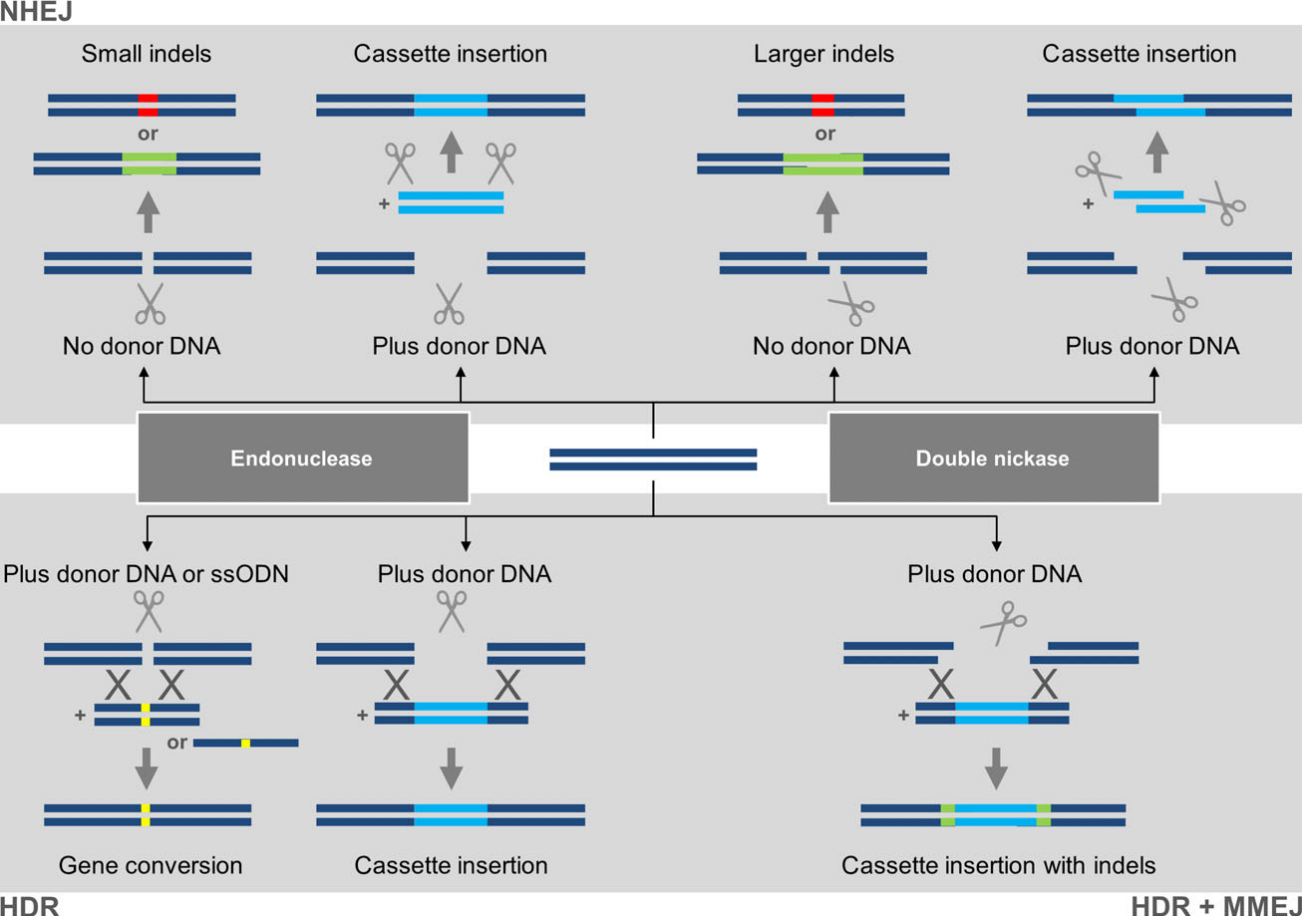
Genome editing with CRISPR/Cas9 can have multiple outcomes depending on the nature of the double‐strand break (DSB), the prevalent repair pathway and the presence of donor DNA. The upper panel shows the major outcomes of the nonhomologous end‐joining (NHEJ) pathway. In the absence of donor DNA, Cas9 endonuclease generates a blunt DSB (indicated by vertical scissors) which is repaired yielding small indels. Alternatively, the double nickase strategy generates a staggered DSB (indicated by diagonal scissors) and these tend to produce larger indels because the single‐stranded tails are often involved in the repair. The indels are shown as insertions (green) or deletions (red). If donor DNA is added to the cell and is flanked by the same target sites present in the genomic locus, then compatible ends are produced which can result in a clean cassette insertion (blue). The lower panel shows the major outcomes of the homology‐dependent repair (HDR) pathway if a donor DNA template is available carrying the desired modification. Donor DNA carrying a subtle change such as a nucleotide substitution (yellow) can be provided as either a duplex molecule or a single‐stranded oligodeoxyribonucleotide (ssODN), and both will lead to allele replacement (gene conversion). Alternatively, the homology region may be used to flank a new sequence which will lead to cassette insertion. If the double nickase approach is used, the single strand overhangs may promote microhomology‐mediated end joining (MMEJ) which can lead to imperfect cassette insertions with indels at the flanks (green).

If Cas9 retains its normal catalytic activity, a blunt DSB is generated at the genomic target site as would be the case in the natural bacterial environment. In higher eukaryotes, the DSB is usually repaired by nonhomologous end joining (NHEJ), an error‐prone pathway that tends to introduce small insertions and deletions, collectively known as indels (Belhaj *et al*., 2013; Quétier, 2016). If no donor DNA is present, these indels are the only footprints of editing and they are often used to trigger frameshift mutations by targeting an exon near the 5′ end of the gene, but if donor DNA is present the same NHEJ events can facilitate the neat insertion of a DNA cassette. These events are shown in the upper left panel of Figure 2. If one of the endonuclease domains is mutated (e.g. RuvC with the D10A mutation or HNH with the H840A mutation), then Cas9 becomes a nickase, and two sgRNAs matching adjacent genomic targets can generate a staggered DSB (Jinek *et al*., 2012; Figure 1b). This increases the accuracy of targeting because specificity relies on two target sites with a total unique sequence length of ~40 bp, and any off‐target nicks are repaired by endogenous repair systems without introducing the errors inherent in DSB repair (Fauser *et al*., 2014; Mikami *et al*., 2016; Ran *et al*., 2013). The other advantage of staggered DSBs is that donor DNA with matching sticky ends can be introduced into the cell or organism, yielding targeting events that facilitate the insertion of a donor DNA cassette (Maresca *et al*., 2013). These events are shown in the upper right panel of Figure 2. As an alternative to dual nickases, staggered breaks can also be introduced with the *Francisella novicida* endonuclease (*Fn*Cpf1), which even in its natural state is a two‐component system (a single gRNA combines the crRNA and tracrRNA functions). It also introduces the DSB (and the resulting indel) at the far end of the target site, thus preserving the original target for subsequent rounds of editing if necessary (Haeussler and Concordet, 2016; Zetsche *et al*., 2015). If both endonuclease domains of Cas9 are mutated, the enzyme becomes catalytically inactive but can still bind at its target site. These dead Cas9 (dCas9) proteins can be used for more diverse applications including epigenetic modification, transcriptional regulation and imaging at the chromosomal level (Hsu *et al*., 2013) or can be fused to a nonspecific endonuclease such as Fok1 as another strategy to generate staggered DSBs (Guilinger *et al*., 2014; Tsai *et al*., 2014; Figure 1c).

The provision of donor DNA can also be used to select for homology‐dependent repair (HDR) events that are common in many microbes but occur once for every 10^5^–10^6^ NHEJ events in higher eukaryotes. This approach can be used either for the insertion of a donor cassette or the replacement of one allele with another, allowing the knockin of entire genes or the replacement of single nucleotides (Belhaj *et al*., 2015). HDR is sometimes resolved cleanly, but in other cases, indels may be formed at the borders of the inserted cassette due to microhomology‐mediated end joining (MMEJ). This is particularly evident when the Cas9 double nickase is used because it leaves single‐stranded tails (McVey and Lee, 2008; Schiml *et al*., 2016). These events are shown in the lower panel of Figure 2. In diploid organisms, targeted mutations can be homozygous, heterozygous or biallelic, the latter resulting from the creation of two different mutant alleles at the target.

## Relevant differences between host species

Many articles have been published describing the use of CRISPR/Cas9 in different species without considering the broader implications and species‐dependent effects caused by target site preference, DSB structure and the characteristics of the target genome. The size of a genome determines the overall number of potential CRISPR/Cas9 targets because the larger the genome, the greater the number of PAMs. However, the larger the number of potential targets the greater the likelihood that some of them will be repeated, so the number of unique targets does not necessarily increase in proportion to genome size. In general terms, monocotyledonous plants (monocots) tend to have larger genomes than dicotyledonous plants (dicots), and vertebrates tend to have larger genomes than invertebrates, although there are many exceptions at the level of individual species (Gregory *et al*., 2007; Li and Du, 2014; Michael and Jackson, 2013). Metazoans tend to have larger genomes than unicellular organisms, and eukaryotes tend to have larger genomes than bacteria and archaea (Li and Du, 2014). However, large variations in genome size within clades of eukaryotes of similar biological complexity indicate variations in the amount of repetitive DNA. Because the CRISPR/Cas9 system is typically used for the editing of genes, the exome is a more relevant comparator than the whole genome. Accordingly, mutation frequencies are generally similar in all animals and plants, suggesting that genome size does not have a significant influence on the efficiency of targeted genome editing mediated by the CRISPR/Cas9 system (Xie and Yang, 2013; Zhang *et al*., 2016).

The GC content of the genome is known to correlate with genome size in bacteria, but in eukaryotes, the relationship is more complex due to the presence of isochores and regions containing highly repetitive DNA (Li and Du, 2014). The GC content varies greatly among different microbes but tends to fall within a relatively narrow range in animals and plants, which is higher in monocots than dicots, and higher in vertebrates than invertebrates. There is also significant variation in GC content among the chromosomes of individual animal and plant species, which is more prevalent in animals than plants, but there is no general correlation between GC content and chromosome size (Li and Du, 2014). The importance of GC content is that it has a significant impact on sgRNA efficiency (Doench *et al*., 2014; Gagnon *et al*., 2014; Ma *et al*., 2015). In plants, animals and microbes, sgRNAs with a GC content greater than 50% are often reported to be more efficient (Feng *et al*., 2014; Jiang *et al*., 2013a,b; Pan *et al*., 2016; Wang *et al*., 2014a; Zhang *et al*., 2014). By analysing sgRNAs that have been experimentally validated in plants, the GC content of most was found to lie between 30% and 80% (Liang *et al*., 2016). Similarly, sgRNAs with an unusually high or low GC content tend to be less effective than those with an average GC content in animals, and sgRNAs targeting the transcribed strand are less effective than those targeting the nontranscribed strand (Wang *et al*., 2014a). The same authors also found that Cas9 preferentially binds to sgRNAs containing purine residues at the last four positions of the spacer sequence and that the efficiency of cleavage is influenced by the affinity between the sgRNA and Cas9. Similar conclusions were drawn during the development of a bioinformatics tool to design sgRNAs for the effective targeting of mouse and human genes (Doench *et al*., 2014). The analysis of sgRNA nucleotide composition in animals has revealed other nucleotide preferences (Doench *et al*., 2014; Wang *et al*., 2014a; Xu *et al*., 2015a), but the same preferences have not been observed in plants (Liang *et al*., 2016). Indeed, no statistically significant preferences in nucleotide composition were observed at any of the 20 positions of the spacer region in plants, suggesting this is a key difference in design principles for sgRNAs used in animals and plants (Liang *et al*., 2016).

## Selection of target sites in different species

### The role of the PAM

The major PAM recognized by *Sp*Cas9 (5′‐NGG‐3′, typically denoted as NGG‐PAM) occurs once every ~10 bp in a random DNA sequence and is found every 8–12 bp in the exons of all host species investigated thus far (Anders *et al*., 2016). Nevertheless, the requirement for this PAM restricts targetable genomic sites to sequences immediately adjacent to this motif. This limitation has been overcome by random mutagenesis followed by screening for mutants with changes in PAM specificity; for example, VRQR‐*Sp*Cas9 and VRER‐*Sp*Cas9 recognize NGA‐PAM and NGCG‐PAM, respectively (Kleinstiver *et al*., 2015). Endonucleases from other sources have also been investigated, such as KKH‐*Sa*Cas9 from *Staphylococcus aureus*, which recognizes N_3_RRT‐PAM (Kleinstiver *et al*., 2015), *Brevibacillus laterosporus* Cas9 (*Blat*Cas9), which recognizes N_4_CND‐PAM (Karvelis *et al*., 2015), and the abovementioned *Fn*Cpf1, which recognizes the unusually AT‐rich TTN‐PAM (Fagerlund *et al*., 2015; Zetsche *et al*., 2015).

The availability of complete genome sequences and oligonucleotide synthesis techniques allows the rapid design and synthesis of sgRNA libraries that can potentially target any gene in the genome (Wang *et al*., 2014a). Many bioinformatics tools are available for the design of sgRNAs (Hendel *et al*., 2015; Mohr *et al*., 2016), and these can often highlight the presence of potential off‐target sites in the genome (Varshney *et al*., 2015). The number of target sequences identified by *in silico* genome analysis is influenced by the stringency of selection; that is, the number and position of any mismatches allowed between the sgRNA spacer and potential off‐target sites. However, indels in the alignment between the target and the sgRNA are not taken into account by most online tools (Lin *et al*., 2014). Although *Sp*Cas9 primarily recognizes NGG‐PAM, it also binds to NAG‐PAM with a much lower affinity, so prediction software can be used to include potential off‐target sites adjacent to either of these motifs. Most tools additionally provide several sgRNA sequences for each gene because the efficiency of targeting depends on many factors, including the uptake/expression of the Cas9 protein and sgRNA, the accessibility of the target and the catalytic efficiency of the enzyme. Recently, Horlbeck *et al*. demonstrated that nucleosomes impede Cas9 binding and cleavage both *in vitro* and *in vivo* in human cells, and they and developed an algorithm to predict highly active sgRNAs taking into account the information on nucleosome occupancy (Horlbeck *et al*., 2016). Several targets may need to be tested to get the best empirical balance between efficient on‐target mutagenesis and the absence of off‐target activity (Bassett *et al*., 2015).

### Selection of targets in plants

An extensive comparative analysis of potential *Sp*Cas9 target sites in plants was carried out by *in silico* prediction to determine the impact of parameters such as genome size and GC content in the dicots Arabidopsis (*Arabidopsis thaliana*), *Medicago truncatula*, soya bean (*Glycine max*) and tomato (*Solanum lycopersicum*) and the monocots *Brachypodium distachyon*, rice (*Oryza sativa*), sorghum (*Sorghum bicolor*) and maize (*Zea mays*; Xie and Yang, 2013; Xie *et al*., 2014). The genome sizes of the eight species ranged from 120 to 2065 Mb which is representative of most land plants, and the GC content ranged from 34% to 47%. One of the outputs of the study was the online database CRISPR‐PLANT (http://www.genome.arizona.edu/crispr/). Potential 20‐bp target sequences were extracted from the current genome sequences and sorted into five different categories according to their specificity and potential for off‐target activity, based on the number and position of mismatches and the presence of NGG‐PAMs and NAG‐PAMs. In all eight species, 5–12 NGG‐PAMs were identified in every 100 bp of the genome and the total number was positively correlated with the genome size. However, the number of specific targets ranged from 4 to 11 million and correlated positively with genome size in dicots but not in monocots. The number of specific targets did not correlate with the number of transcripts or with the number of NGG‐PAMs. These results indicated that although larger genomes contain more PAMs and therefore more potential targets, the new targets were more likely to align with others and would therefore lack specificity. For seven of the eight species, it was possible to design specific sgRNAs to target 83%–99% of annotated transcripts (94.3% for Arabidopsis, 83.4% for *M. truncatula*, 89.5% for tomato, 96.4% for soya bean, 98.6% for *B. distachyon*, 87.3% for rice and 92.6% for sorghum) and 67.9%–96% of these transcripts contained at least 10 different targetable NGG‐PAM sites. This indicated that off‐target effects are unlikely to present a constraint in most plant species. The exception was maize, where only 29.5% of annotated transcripts matched a specific sgRNA. Among the eight species, maize had the largest genome, the highest GC content and the greatest number of annotated transcripts, reflecting the abundance of highly repetitive DNA and dispersed repeats. These features are shared with other cereals such as wheat and barley, and it may be challenging to develop unique target sites for the majority of genes in these species, although genome editing using CRISPR/Cas9 has been successful in all three cereals (Lawrenson *et al*., 2015; Svitashev *et al*., 2015; Upadhyay *et al*., 2013). Cas9 variants, such as the abovementioned *Sp*Cas9 VQR and VRER mutants that recognize noncanonical PAMs, can broaden the range of genome editing; that is, they approximately double the number of accessible sites in rice compared to wild‐type *Sp*Cas9 and may therefore facilitate the editing of more complex plant genomes (Hu *et al*., 2016).

### Selection of targets in other eukaryotes

Two independent studies have considered the creation of genome‐scale sgRNA libraries for human cells, which can be considered a model for mammals and perhaps vertebrates in general due to the overall similarity in broad genomic characteristics. A library comprising 73 151 sgRNAs targeting 7114 genes (preferentially constitutive 5′ exons) and 100 nontargeting controls was designed by Wang *et al*. (2014a), and the sgRNAs were filtered for potential off‐target effects based on sequence similarity, the presence of at least two mismatches compared to any off‐target site, a GC content of 20%–80% (to reduce the likelihood of secondary structures) and fewer than four consecutive identical nucleotides. The library included 10 sgRNAs for each of 7033 protein‐coding genes as well as all possible sgRNAs for each of the 84 genes encoding ribosomal proteins. Shalem *et al*. (2014) tested the feasibility of genome‐scale CRISPR/Cas9 knockout screening by designing and testing a library of 64 751 sgRNAs targeting the constitutive 5′ exons of 18 080 genes with an average coverage of 3–4 sgRNAs per gene. Each target site was selected with a refined heuristic approach to minimize off‐target activity. A zebrafish sgRNA library was developed based on 18 367 469 target sequences predicted in the reference genome, allowing the rapid selection of sgRNAs with embedded information concerning the predicted off‐target sites for each 12‐bp seed region (Varshney *et al*., 2015). Each 20‐bp target site adjacent to an NGG‐PAM site differs by at least three mismatches from any other target sequence adjacent to an NGG‐PAM or NAG‐PAM. A genome‐wide sgRNA library for Drosophila (*Drosophila melanogaster*) has been prepared containing 40 279 sgRNAs targeting 13 501 genes (78% of all genes) including 8989 targeted by three or more independent sgRNAs (Bassett *et al*., 2015) and the *Saccharomyces cerevisiae* genome contains 645 392 specific targets (unique seed region followed by a NGG‐PAM) and 108 493 less specific targets (DiCarlo *et al*., 2013).

## Comparison of targeting parameters

### Resolution and targeting efficiency in plants

The resolution of gene editing refers to the nature of the repair pathway (NHEJ, MMEJ and/or HDR) and the broad definition of the resulting mutation (insertion, deletion, replacement, inversion translocation) as shown in Figure 2. NHEJ is the preferred DNA repair pathway in somatic plant cells (Puchta, 2005); therefore, most CRISPR/Cas9 events are resolved by this mechanism, resulting in error‐prone repair and the introduction of indels. The reported efficiency of indels induced by CRISPR/Cas9 in both dicots and monocots can vary significantly even within the same species (e.g. 1.1%–90.4% in Arabidopsis), but most species offer examples of efficiency approaching 100%, including Arabidopsis (Yan *et al*., 2015), soya bean (Cai *et al*., 2015), potato (Wang *et al*., 2015a), tomato (Pan *et al*., 2016), petunia (Zhang *et al*., 2016), tobacco (Gao *et al*., 2015), poplar (Zhou *et al*., 2015), grapefruit (Jia *et al*., 2016), maize (Svitashev *et al*., 2015) and rice (Ma *et al*., 2015). Notably, the CRISPR/Cas9 system can efficiently induce mutations in different tissues and cell types, including embryogenic callus (e.g. rice and maize), hairy roots (e.g. soya bean), protoplasts (e.g. *Nicotiana benthamiana*, lettuce, maize and rice), cotyledons (e.g. tomato) and leaves (e.g. *N. benthamiana*, petunia and poplar). Studies reporting the efficiency of different types of targeting events in plants are summarized in Table S1. This intraspecific variability partly reflects how our knowledge of the CRISPR/Cas9 system has increased since the system was first used for genome editing, resulting in more efficient experimental designs. Although direct comparisons between experiments carried out under different conditions are not possible, two major common principles have emerged.

The first principle is that the efficiency of genome editing is strongly influenced by the expression of the components. In Arabidopsis, initial experiments based on *in planta* transformation and Cas9 expression controlled by the constitutive *Cauliflower mosaic virus* (CaMV) 35S promoter resulted in low editing efficiencies and mostly somatic mutations that were not transmitted to the progeny. But the frequency of heritable mutations increased to 90.4% when the constitutive promoter was replaced with an egg‐cell‐specific promoter (Wang *et al*., 2015b), a cell‐division‐specific promoter (Hyun *et al*., 2015; Yan *et al*., 2015) or a germ‐line‐specific promoter (Mao *et al*., 2016). In all other plant systems, constitutive promoters have achieved high mutation frequencies, with biallelic and homozygous mutants readily produced in the first generation of monocots such as maize (Svitashev *et al*., 2015) and rice (Lowder *et al*., 2015; Ma *et al*., 2015; Miao *et al*., 2013; Wang *et al*., 2016) and dicots such as tomato (Brooks *et al*., 2014; Pan *et al*., 2016), poplar (Fan *et al*., 2015; Zhou *et al*., 2015), potato (Wang *et al*., 2015a), petunia (Zhang *et al*., 2016) and tobacco (Gao *et al*., 2015). These observations also suggest that mutagenesis often occurs at an early stage during the transformation process, before the first cell division. High levels of sgRNA limit the efficiency of genome editing at least in tomato (Pan *et al*., 2016) and Arabidopsis (Ma *et al*., 2015). In comparison with protoplasts, the efficiency of targeted chromosomal fragment deletion between paired sgRNA/Cas9 sites is lower in transgenic plants. This may reflect the relatively low levels of sgRNAs and Cas9 in callus tissue and regenerated plants (Xie *et al*., 2015).

The second principle is that the nature of the sgRNA is also an important determinant of targeting efficiency. Although the rules are not completely understood, there is little doubt that some sgRNAs are more mutagenic than others and this is a key factor in determining the outcome of each editing experiment. As stated above, sgRNAs with a GC content greater than 50% often have a high editing efficiency, possibly because of a stronger binding to their target site (Feng *et al*., 2014; Jiang *et al*., 2013a; Pan *et al*., 2016; Wang *et al*., 2014a; Zhang *et al*., 2014). In addition to the GC content of the spacer, the secondary structures of sgRNAs also affect the efficiency of editing (Makarova *et al*., 2011). The formation of a stem‐loop structure in the protospacer region can inhibit the binding of the sgRNA to the target strand, reducing the likelihood of a DSB (Ma *et al*., 2015). Targeting one gene with multiple sgRNAs has been shown to greatly increase the mutation frequency and the recovery of homozygous mutants in rice (Wang *et al*., 2016; Xie *et al*., 2015; Zhang *et al*., 2014) and T0 tomato plants (Brooks *et al*., 2014). An extension of the culture period increased the proportion of mutated cells in *Agrobacterium tumefaciens*‐infected rice callus (Mikami *et al*., 2015a,b) and in soya bean somatic embryo cultures (Jacobs *et al*., 2015), probably reflecting the proliferation of existing mutant cells as well as new mutations. However, this method can also reduce the regeneration capacity of the cells and increase the risk of obtaining chimeric plants (Xu *et al*., 2015b).

The efficiency of genome editing by HDR is generally lower than NHEJ because homologous recombination occurs 10^5^–10^6^ times less frequently than repair by end ligation in plants (Figure 2). Only a handful of reports describe successful genome editing by HDR in higher plants, but they represent an interesting variety of different approaches. Gene conversion was achieved with an efficiency of 5% using short single‐stranded oligodeoxyribonucleotides (ssODNs) as the repair template in Arabidopsis (Sauer *et al*., 2016) and with an efficiency of 9% using dsDNA as the repair template in *N. benthamiana* (Li *et al*., 2013). When directly compared in maize, a short ssODN template (127 nt) was twice as efficient as a plasmid donor (800 bp), achieving mutation frequencies of 0.4% and 0.2%, respectively (Svitashev *et al*., 2015). Expression cassettes flanked by 1‐kb homology arms were inserted with a frequency of 4.6% in soya bean (Li *et al*., 2015) and 4% in maize (Svitashev *et al*., 2015). Gene replacement using a combinatorial dual‐sgRNA/Cas9 vector to remove 255 bp of endogenous sequences and insert a ~1.9‐kb cassette with homology arms of 733 and 825 bp was achieved with a frequency of 0.8% in Arabidopsis (Zhao *et al*., 2016). In one of the first examples of CRISPR/Cas9 genome editing, Li *et al*. (2013) failed to induce HDR in Arabidopsis protoplasts and attributed this to the intrinsically low efficiency of HDR in these cells compared to *N. benthamiana*, but more recent data suggest that the low efficiency probably reflected the relatively small number of DSBs, given that a 1.1%–5.6% mutation frequency was achieved by NHEJ with the same sgRNAs. An outstanding efficiency of 100% HDR‐mediated conversion of the *ALS* gene was achieved in rice by Sun *et al*. (2016) using two sgRNAs for cleavage, flanking the homology arms on the donor with CRISPR sites to release the repair template *in vivo* and increasing the amount of donor DNA by introducing both the vector donor and free donor fragments (476 bp). This is by far the highest HDR frequency observed in higher eukaryotes but has yet to be tested in other species, and it will be necessary to determine whether undesirable random integration events also occur in this system. Particle bombardment appears to be up to fivefold more effective than *Agrobacterium*‐mediated transformation for the promotion of HDR induced by CRISPR/Cas9 in maize (Svitashev *et al*., 2015) and rice (Sun *et al*., 2016). Lower levels of Cas9 but higher levels of sgRNA and repair template can increase the likelihood of resolution by HDR in yeast (Stovicek *et al*., 2015), and this has been achieved by particle bombardment in rice (Sun *et al*., 2016) and viral replicons in tobacco (Baltes *et al*., 2014). Interference with the NHEJ pathway can also promote HDR as demonstrated in rice using a *lig4* mutant background to abolish end‐joining ligase activity, although the impairment of NHEJ may also increase the frequency of spontaneous mutations (Endo *et al*., 2016).

### Resolution and targeting efficiency in animals

**Figure 3:**
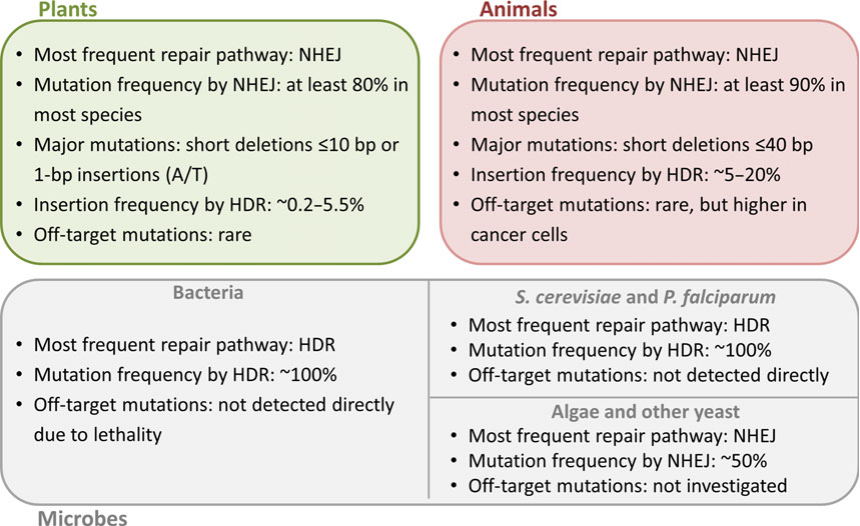
The outcome of genome editing with CRISPR/Cas9 is subject to species‐dependent effects determined by the prevalent DNA repair pathways. Nonhomologous end joining (NHEJ) is prevalent in plants and animals, but the resulting indels tend to be smaller in plants than animals, and 1‐bp insertions of A/T pairs are moderately frequent in plants but unusual in animals. Animal cells are also more efficient at HDR than plant cells although the frequency depends on the species and cell type. The range of HDR‐based insertion frequencies in plants represents a broad analysis of eight articles reporting such data, but exceptional HDR frequencies as high as 9% (Li *et al*., 2013) and 100% (Sun *et al*., 2016) have been reported, the latter achieved by including additional free donor fragments. In contrast to animals and plants, NHEJ is much less prevalent in microbes generally, but particularly in bacteria and certain eukaryotes (including the yeast *Saccharomyces cerevisiae* and the malaria parasite *Plasmodium falciparum*). In these species, NHEJ products are so rare that CRISPR/Cas9 without a donor template is often lethal and can be used to select for HDR events without using marker genes. In other yeast and in algae, the NHEJ pathway is prevalent and the behaviour is likely to be similar to that observed in animals and plants. These principles were derived by the authors from the data in Tables S1–S3.

The CRISPR/Cas9 system has been used successfully in many animals, including invertebrates and vertebrates (Table S2). Unlike plants, the CRISPR/Cas9 system can be used for genome editing in particular tissues by hydrodynamic injection or by introducing the components using *Adeno‐associated virus* (AAV) or *Adenovirus* vectors (Rodriguez *et al*., 2014; Senis *et al*., 2014; Swiech *et al*., 2015; Xue *et al*., 2014). Although there are differences in DNA repair pathways across species, the general preponderance of NHEJ over HDR observed in plants is also observed in animals (Figure 3). Indels generated by NHEJ have therefore been introduced with an efficiency of up to ~90% in the nematode *Caenorhabditis elegans* (Friedland *et al*., 2013), Drosophila (Bassett *et al*., 2015), rabbit (Lv *et al*., 2016), chicken (Oishi *et al*., 2016), mouse (Yang *et al*., 2014) and human cells (Liang *et al*., 2015), whereas the insertion of donor DNA by HDR has been reported with a frequency of ~5%–20% in *C. elegans* (Dickinson *et al*., 2013), rat (Shao *et al*., 2014) and mouse (Platt *et al*., 2014). Site‐specific indels were induced in zebrafish embryos by the *in vivo* microinjection of a sgRNA/Cas9 complex incorporating an additional tracrRNA sequence, causing mutagenesis at two sites that were impossible to edit with TALENs (Hwang *et al*., 2013). In mouse, the use of ssODNs instead of a plasmid donor increased the efficiency of HDR from 10%–30% to 10%–80% (Yang *et al*., 2014).

In human cell lines, reported mutation frequencies are often significantly lower than 5%, especially when induced pluripotent stem cells are used as the host (Miyaoka *et al*., 2016; Yang *et al*., 2013; Zhu *et al*., 2015). However, much higher mutation rates of ~60–90% have also been reported in these cells, which may reflect differences in the activity of specific sgRNAs (Liang *et al*., 2015; Veres *et al*., 2014). Gene knockin by HDR in human cells is usually successful in fewer than 10% of cells, although this can be increased to 50%–66% if NHEJ is suppressed (Chu *et al*., 2015). The transfection of human stem cells expressing a doxycycline‐inducible Cas9 gene (iCas9) with sgRNAs enabled the drug‐free selection of precise HDR‐mediated modifications with ssODN donors (Zhu *et al*., 2015). The efficiency of HDR also increased sevenfold when short hairpin RNAs were introduced expressing the *Adenovirus* Ad4 protein responsible for suppressing Ligase 4, which is required for NHEJ in mice and humans (Chu *et al*., 2015). Biallelic double‐gene mutants were generated by transfecting cells with three separate plasmids and injecting ssODNs matching two of the target genes to achieve HDR‐mediated mutation (Wang *et al*., 2013). Mice were engineered to constitutively express Cas9, and when injected with a combination of sgRNAs and HDR donors carried by AAV vectors, NHEJ and HDR events occurred at frequencies that increased over time (Platt *et al*., 2014).

### Resolution and targeting efficiency in microbes

The CRISPR/Cas9 system is a double‐edged sword in bacteria because DSBs are inefficiently repaired by NHEJ, thus targeting the bacterial genome with CRISPR/Cas9 tends to be lethal (Selle and Barrangou, 2015). However, most bacterial strains are either recombinogenic or have established functional recombineering systems, so the provision of donor DNA allows the CRISPR/Cas9 system to be used as a powerful tool for the selection of HDR events and the simultaneous counterselection of background cells, resulting in highly efficient HDR without the need for positive and negative selectable markers. Using this strategy, the wild‐type *Sp*Cas9 system has been applied in both Gram‐positive and Gram‐negative bacteria with an HDR efficiency of 65%–100% (Table S3). In *Clostridium cellulolyticum*, the Cas9 nickase variant together with a repair template achieved HDR efficiencies greater than 95% (Xu *et al*., 2015c).

Eukaryotic microbes show diverse behaviours in response to CRISPR/Cas9, and the system must be tailored for use in different species. In contrast to higher plants, the constitutive expression of Cas9 is toxic in *Chlamydomonas reinhardtii*, preventing the recovery of transformants even in the absence of sgRNA. The basis of this phenomenon is unclear, but the transient expression of Cas9 and sgRNA has proven sufficient to generate indels by NHEJ (Jiang *et al*., 2014) as has the delivery of Cas9 RNPs (Shin *et al*., 2016). In contrast, Cas9 is not toxic in the marine diatom *Phaeodactylum tricornutum* and mutation frequencies of up to 63% have been achieved by NHEJ following stable transformation with a *cas9* transgene (Nymark *et al*., 2016). Similarly, different yeast species show diverse behaviours in response to genome editing. *S. cerevisiae* is unusually amenable to HDR, so gene editing at the single nucleotide level can be achieved using short donor templates (Bao *et al*., 2015; Biot‐Pelletier and Martin, 2016; DiCarlo *et al*., 2013) and the simultaneous deletion and insertion of several genes has been reported at frequencies of up to 100% (Stovicek *et al*., 2015; Tsai *et al*., 2015a). Although targeted nicks and DSBs increase the HDR frequency by up to 4000‐fold in *S. cerevisiae*, only a 10‐fold increase was observed in *Pichia pastoris*, where the NHEJ pathway is more prevalent (Weninger *et al*., 2016). The malaria parasite *Plasmodium falciparum* is an interesting target because it appears to be naturally deficient in the canonical NHEJ pathway, and only HDR occurs when a donor template is provided (Ghorbal *et al*., 2014). Genome editing using the CRISPR/Cas9 systems therefore relies on DSB‐induced HDR with an external donor template, and this has been achieved with an efficiency of 50%–100% when using ssODNs.

## Mutation signatures

The mutations induced by CRISPR/Cas9 in plants are mainly short deletions of 10 bp or less and single‐base insertions, typically A/T in all species (Figure 3). Single‐base substitutions are rare, with the exception of soya bean protoplasts where they were the most frequent mutation (Sun *et al*., 2015). Less frequent longer deletions may represent the results of MMEJ, indicating that gene‐specific factors can influence the outcome of DSB repair (Xu *et al*., 2015b). In rice, mutation signatures vary according to the target (Miao *et al*., 2013; Xu *et al*., 2015b; Zhou *et al*., 2014). The consistent mutations observed at the same target in several independent soya bean hairy‐root cultures and somatic embryos also suggest that there may be as yet undiscovered rules governing the types of mutations that are favoured at a given target (Jacobs *et al*., 2015). In the same study, the seven most effective sgRNAs exclusively generated short deletions, whereas those with lower efficiency were associated with more insertions and substitutions (Jacobs *et al*., 2015). Interestingly, all the off‐target mutations found in rice by Li *et al*. (2016) were 1‐bp insertions, indicating that the pairing of sgRNA with the target sequence may also influence the mutation type.

The picture emerging from studies in animal systems is similar to that in plants: most on‐target and off‐target mutations reported in animals are short deletions of up to 40 bp (An *et al*., 2016; Friedland *et al*., 2013; Kim *et al*., 2015). Although less common, deletions as large as 250 bp have been reported in mice (Heckl *et al*., 2014) and human cells (Liang *et al*., 2015), but insertions are usually shorter, typically 1–15 bp (Cheng *et al*., 2014; Friedland *et al*., 2013; Kim *et al*., 2015; Liang *et al*., 2015). CRISPR/Cas9 mutation signatures are not widely reported in microbes due to the lethality of the NHEJ pathway in bacteria and some microbial eukaryotes, and the relatively small number of studies.

The Cas9 double nickase generates mutations with different signatures compared to the intact enzyme because of the nature of the staggered DSB. For example, a Cas9 double nickase was used to generate a DSB with 52‐nt overhangs in Arabidopsis. The average size of the resulting insertions was considerably larger (>80 nt) than for the fully functional Cas9 nuclease, and in most cases the insertions were copies of the sequence immediately upstream or downstream from the insertion site, which is indicative of MMEJ (Schiml *et al*., 2014, 2016). The double nickase approach also generated longer deletions in mice (39–56 bp) as well as insertions of up to 67 bp (Cheng *et al*., 2014). Interestingly, the two alternative Cas9 variants from *S. thermophilus* and *S. aureus* generate different mutation signatures associated with different PAMs in Arabidopsis. For *St*Cas9, sgRNAs with a NNAGAA‐PAM generated 8.6% insertions (mostly 1‐bp) and 3.8% deletions, whereas those with a NNGGAA‐PAM generated 11.6% insertions (mostly 1‐bp) and 3.8% deletions. For *Sa*Cas9, sgRNAs with a NNGAA‐PAM generated 52.1% insertions (mostly 1‐bp), whereas those with a NNGGGT‐PAM generated 46.7% deletions and 21.6% insertions, both of which were generally larger than 1 bp (Steinert *et al*., 2015).

## Off‐target mutations and methods to increase efficacy and accuracy

### Off‐target mutations in plants

Off‐target activity is generally rare in higher plants (Table S1). Where reported, it tends to involve a minority of sgRNAs even when mutations are investigated by the thorough method of whole‐genome sequencing (Feng *et al*., 2014). A low frequency of unwanted mutations has been reported when the sgRNA features mismatches outside the seed sequence, for example in rice (Xu *et al*., 2015b; Zhang *et al*., 2014) and wheat (Upadhyay *et al*., 2013), indicating that such events could be avoided by designing more specific sgRNAs. Careful sgRNA design can ensure specific targeting even when the genome contains closely related paralogous genes (Baysal *et al*., 2016). However, in a small number of cases, unexpected cleavage has been observed at sites with one or more mismatches within the seed region, for example in Arabidopsis (Sauer *et al*., 2016), barley (Lawrenson *et al*., 2015), soya bean (Jacobs *et al*., 2015) and rice (Xie and Yang, 2013). Target sequences with a GC content higher than 70% may increase the likelihood of off‐target effects (Tsai *et al*., 2015b), which might explain the unexpected mutations observed by Li *et al*. (2016) (GC = 65%–80%) but not those reported by Jacobs *et al*. (2015) (GC = 57%) or Sauer *et al*. (2016) (GC = 50%). Even so, the frequency of off‐target mutations is much lower than that of on‐target mutations, allowing the recovery of solely on‐target mutations in all experiments. Interestingly, Xu *et al*. (2015b) detected off‐target mutations only in T1 rice plants carrying the *cas9* and *sgRNA* transgenes, but not in those where the CRISPR components had segregated, suggesting that off‐target effects might be reduced or avoided by selecting appropriate T1 progeny. The frequency of unwanted mutations depends on the abundance of the Cas9/sgRNA RNP complex so the likelihood can be reduced by transient expression of the components rather than stable transgene integration, although this could reduce on‐target efficiency too (Tsai *et al*., 2015b). The Cas9 double nickase resulted in efficient genome engineering in Arabidopsis, without off‐target effects in homologous genomic regions (Fauser *et al*., 2014). On the whole, off‐target mutations in plants are generally less frequent than the somatic mutations that arise during tissue culture (Li *et al*., 2016).

### Off‐target mutations in animals and microbes

Off‐target mutations in human cells were initially reported to be up to 50% more common than mutations at the on‐target site, raising concerns about the intrinsic fidelity of the CRISPR/Cas9 system (Fu *et al*., 2013; Hsu *et al*., 2013; Kim *et al*., 2015; Mali *et al*., 2013; Pattanayak *et al*., 2013). However, those experiments were conducted on cancer cell lines, which are often characterized by dysfunctional DNA repair mechanisms, or used sgRNAs that are known to be promiscuous (Kim *et al*., 2015). When stem cells were used as the host, whole‐genome sequencing revealed the absence of off‐target mutations (Smith *et al*., 2014) or only a few off‐target events (Veres *et al*., 2014). Similarly, minimal off‐target activity has been reported in zebrafish (Hruscha *et al*., 2013), mice (Heckl *et al*., 2014), chicken (Oishi *et al*., 2016) and rabbit (Lv *et al*., 2016). These studies are summarized in Table S2. However, for both animals and plants, most studies have sought off‐target activity at preselected sites rather than by unbiased whole‐genome sequencing, which means that off‐target activity cannot be ruled out at unpredicted sites. Off‐target mutations have not been directly observed in microbes but may be inferred due to their indirect toxicity (Table S3).

## Methods to increase targeting efficiency and accuracy

Several approaches have been described to increase the efficiency and accuracy of CRISPR/Cas9 in plants, animals and microbes regardless of the host species and tissue, based on sgRNA design, nuclease choice and the delivery strategy. The ideal sgRNA should maximize on‐target activity while minimizing off‐target activity. In addition to following the general principles of sgRNA design discussed above, fidelity can be improved using a truncated spacer (<20 nt) and by adding two guanidine residues to the 5′ end of the sequence (Cho *et al*., 2014; Fu *et al*., 2013; Kim *et al*., 2015). The choice of nuclease can also increase targeting accuracy. Cas9 nickases must act as dimers and therefore double the length of the target site, reducing off‐target activity by 50‐ to 1500‐fold (Fauser *et al*., 2014; Ran *et al*., 2013; Schiml *et al*., 2014). This is also true for the hybrid endonuclease dCas9‐FokI which is generated by fusing the enzymatically inactive dCas9 to the nonspecific endonuclease domain of the restriction enzyme FokI (Guilinger *et al*., 2014; Tsai *et al*., 2014). The propensity of Cas9 to tolerate mismatches between the sgRNA and target site is attributed to a high binding energy, so the rational engineering of amino acid residues involved in DNA binding has produced two high‐fidelity mismatch‐sensitive variants of Cas9 that achieved promising results in human cells (Kleinstiver *et al*., 2016; Slaymaker *et al*., 2016). The delivery of the sgRNA, nuclease and (where appropriate) the HDR donor molecule also affects the outcome of the experiment. Typically, the components are delivered as plasmid DNA and must be expressed in the cell, but they can also be introduced as RNA or as preformed RNP complexes that can be delivered efficiently by electroporation or transfection to mammalian cells (Burger *et al*., 2016; Chu *et al*., 2016; Liang *et al*., 2015), plant protoplasts (Subburaj *et al*., 2016; Woo *et al*., 2015) or microalgae (Shin *et al*., 2016). Because the RNP complexes are cleared rapidly, they are less likely to cleave at off‐target sites (Liang *et al*., 2015). For HDR, the use of short ssODN donor molecules seems to result in higher insertion frequencies than donor delivery by plasmid, but the size is limited to ~200 nt (Sauer *et al*., 2016; Zhao *et al*., 2016). Like Cas9, endonucleases from the Argonaute family have recently been found also to use oligonucleotide guides to target invasive genomes (Gao *et al*., 2016). The DNA‐guided nuclease NgAgo binds a 5′‐phosphorylated, single‐stranded, ∼24‐nt guide DNA (gDNA) that creates site‐specific DSBs without needing a PAM. Preliminary characterization suggests a low tolerance of gDNA/target mismatches and highly efficient editing of GC‐rich genomic targets. However, the efficiency of the system and reproducibility of the results obtained with NgAgo are being questioned (Cyranoski, 2016), so it remains to be seen whether it can be effectively used for genome editing.

## More ambitious genome editing

### Multiplex targeting

One of the features of the CRISPR/Cas9 system that sets it apart from ZFNs and TALENs is that multiple sgRNAs can be introduced simultaneously into a cell with little additional effort, allowing more ambitious genome‐editing strategies such as the simultaneous mutation of different genes and the creation of more extensive mutations. Multiple genes can be targeted with one sgRNA by deliberately designing a promiscuous sequence, and this has been used to mutate multiple targets in related rice genes due to the tolerance of mismatches (Endo *et al*., 2015). Multiple sgRNAs can be introduced either as separate constructs or in the form of a polycistronic cassette, which allows any number of targets to be edited simultaneously regardless of their relationship (Xie *et al*., 2015). The latter strategy has been used extensively in rice, for example to generate null alleles at the *SIAGO7* locus (Brooks *et al*., 2014), to mutate multiple genes at the *YSA* locus (Lowder *et al*., 2015), to simultaneously mutate multiple genes in the *MPK* family (Xie *et al*., 2015), to investigate the frequency of mutations in the *ERF922* gene (Wang *et al*., 2016) and to mutate the *GSTU* and *MRP15* genes in rice and Arabidopsis (Ma *et al*., 2015). Gao *et al*. (2015) simultaneously mutated the tobacco *PDS* and *PDR6* genes which generate an albino phenotype. Multiple genes or multiple targets within genes have also been mutated in animals. For example, Dickinson *et al*. (2013) simultaneously mutated four targets in the *C. elegans lin‐31* gene to modify the MAP kinase phosphorylation sites at the C‐terminus. Platt *et al*. (2014) simultaneously mutated the mouse *TP53*,* LKB1* and *KRAS* genes, which are the three most prevalent oncogenes in the lung, and a similar approach was used to study functional redundancy within the *TET* gene family (Wang *et al*., 2013).

### Large‐scale mutations

Multiple sgRNAs not only allow simultaneous targeting at different sites but they can also be combined to induce large deletions and other rearrangements. For example, chromosomal segments of up to 245 kb have been deleted in rice plants by the introduction of two sgRNAs that create DSBs on the same chromosome (Zhou *et al*., 2014), and similar strategies have been used to achieve deletions ranging from 65 kb to 30 Mb in mammalian cells (Essletzbichler *et al*., 2014; Zhang *et al*., 2015). Whereas most tandem DSBs are resolved by deleting the intervening DNA, another possible outcome is the creation of a chromosomal inversion. Li *et al*. (2015) reported that precise inversions of DNA fragments ranging in size from ~50 bp to hundreds of kb could be generated efficiently in mice and human cells, as well as deletions and duplications resulting from transallelic recombination between DSBs on sister chromatids. Similarly, large‐scale rearrangements have been generated in human cell lines to model the chromosomal hallmarks of cancer. For example, Choi and Meyerson (2014) used pairs of sgRNAs to introduce paracentric and pericentric chromosomal inversions as well as the *CD74‐ROS1* chromosomal translocation event often seen in lung cancer, and Maddalo *et al*. (2014) used a similar strategy to induce the *Eml4‐Alk* inversion which is a hallmark of non‐small‐cell lung carcinoma. Chromosomal translocations resembling those associated with acute myeloid leukaemia and Ewing's sarcoma were induced at a high frequency using pairs of sgRNAs targeting different chromosomes by Torres *et al*. (2014). In contrast, translocations have not yet been reported in plants, and chromosomal inversions are rare: Liang *et al*. (2016) found that only one of nine expression constructs producing sgRNA pairs was able to generate an inversion and such events occurred in only two of the 23 transgenic rice plants expressing this sgRNA pair. Interestingly, Li *et al*. (2015) reported that most chromosomal inversions in their human and mouse cell lines were accompanied by small terminal indels, suggesting that repair was promoted by MMEJ. The difference in the frequency of inversions and other chromosomal rearrangements between mammals and plants may therefore reflect the relative activity of this repair pathway.

## Editing targets currently in commercial development

In addition to proof‐of‐concept and optimization studies in model systems, there is now great interest in the commercial applications of CRISPR/Cas9, particularly in the pharmaceutical industry where it can be used to develop accurate disease models and platforms for drug screening (Jang and Ye, 2016; Tschaharganeh *et al*., 2016), and in the agricultural industry where it can be used to produce new crop and farm animal varieties with enhanced traits (Sovová *et al*., 2016). The need to characterize the outcome of genome editing in detail as a way to facilitate commercial development is therefore clear, not only to streamline the development of innovative products but also in the light of the different regulatory pathways for genome‐edited crops in the USA (based on product, considered an output of plant breeding) and the EU (based on process, considered GMO technology at the moment but still under debate; Sprink *et al*., 2016). In the field of plant biotechnology, research is now focusing on genome editing in a broad range of crop species including citrus fruits, maize, poplar, potato, rice, sorghum, soya bean, tomato and wheat (Khatodia *et al*., 2016; Song *et al*., 2016). The purpose of such studies is the improvement of agronomic traits, but most publications describe the ability to edit the relevant target genes rather than the traits themselves (Sovová *et al*., 2016). For example, CRISPR/Cas9 has been used to knock out the rice *sweet* genes that confer sensitivity to bacterial blight (Jiang *et al*., 2013b), the wheat *MLO* genes that confer sensitivity to powdery mildew (Shan *et al*., 2013; Wang *et al*., 2014b), and the rice *mpk5* gene that regulates defence responses (Xie and Yang, 2013). Although all these studies were successful, some were conducted on protoplasts, and even when plants were regenerated they were not tested directly for pathogen resistance. In contrast, cucumber plants in which the *elF4E* gene encoding eukaryotic translation initiation factor 4E was mutated using CRISPR/Cas9 have been tested for resistance against *Cucumber vein yellowing virus*,* Zucchini yellow mosaic virus* and *Papaya ring spot mosaic virus* (Chandrasekaran *et al*., 2016). Similarly, Wang *et al*. (2016) knocked out the rice *OsERF922* gene encoding the transcription factor ERF and showed that the homozygous T2 plants were more resistant to rice blast but were otherwise identical to wild‐type plants in terms of growth and yield traits. Sun *et al*. (2016) used CRISPR/Cas9 to induce HDR in rice, resulting in a single nucleotide substitution in the *ALS* gene that conferred herbicide resistance. Li *et al*. (2016) individually mutated four rice genes affecting yield traits (*Gn1a*,* DEP1*,* GS3* and *IPA1*) and achieved a variety of promising mutant phenotypes in the T2 generation, including more grains (*gn1a*), denser panicles and semi‐dwarf culms (*dep1*), larger grains and long awns (*gs3*), and a change in tiller number, either more or less depending on the precise target site (*ipa1*). Finally, CRISPR/Cas9 has also been used to target genes encoding polyphenol oxidases (PPOs) in mushroom. The enzyme causes browning of the fungal tissue, and knocking out one of six genes in the PPO gene family reduced overall PPO activity by 30% thus extending the shelf life (Waltz, 2016).

In academic research, the choice of genome‐editing technique depends mainly on the simplicity and cost of the approach and the availability of tools and expertise, but applied research and commercial crop development must also take into account the associated intellectual property (IP) and licensing issues. Each of today's genome‐editing tools is protected by patents or patent applications (Schinkel and Schillberg, 2016), and navigation of the IP landscape is straightforward in the case of oligonucleotide‐directed mutagenesis, ZFNs and TALENs (Table S4). In contrast, the IP situation for the CRISPR/Cas9 technology is strongly contested by at least three major players: Massachusetts Institute of Technology/Broad Institute, UC Berkeley and Vilnius University (Schinkel and Schillberg, 2016). The ongoing legal dispute has delayed the commercial development of crops produced using CRISPR/Cas9 technology, although DuPont Pioneer has recently received an exclusive licence (Grushkin, 2016). DuPont Pioneer has exploited CRISPR/Cas9 technology for the development of drought‐resistant maize and waxy maize with an improved starch composition. In the latter case, CRISPR/Cas9 was used to knock out the *Wx1* gene resulting in maize kernels that only accumulate amylopectin. The company recently announced that they will bring the genome‐edited maize to the market within the next 5 years.

## Summary and outlook

The CRISPR/Cas9 system has been used for genome editing in a wide range of different organisms but the outcome in terms of resolution, efficiency, accuracy and mutation structure depends on various factors including target site choice, sgRNA design, the properties of the endonuclease, the type of DSB introduced, whether or not the DSB is unique, the quantity of endonuclease and sgRNA, and the intrinsic differences in DNA repair pathways in different species, tissues and cells. Species‐dependent effects include the preponderance of NHEJ compared to HDR in higher eukaryotes, contrasting with the preference for HDR in bacteria and some unicellular eukaryotes, and subtle differences in the mutation signatures generated in animals and plants (Figure 3). Whereas canonical CRISPR/Cas9 predominantly introduces small deletions (<10 bp) and single‐base insertions in plants, both types of indel tend to be larger in animals (deletions <40 bp and insertions of 1–15 bp) and there is a greater frequency of larger deletions. In both animals and plants, Cas9 double nickase introduces staggered DSBs and this results in even larger indels (typically <100 bp). Another difference is the relative efficiency of larger genome rearrangements in animals compared to plants. These differences are likely to reflect species‐dependent aspects of the competing NHEJ, MMEJ and HDR repair pathways, suggesting that the outcome of genome editing could be influenced by modulating the activity of particular repair enzymes, as shown by the increased prevalence of HDR in cells lacking normal levels of Ligase 4 in both animals and plants. Further investigations and detailed comparisons of genome‐editing outcomes in different species will provide insight into interaction between component‐specific effects (nuclease activity, sgRNA design) and host‐specific effects (genome structure and content, DNA repair pathways) to enable the refinement of genome‐editing strategies in a context‐dependent manner.

## Conflict of Interest

Fraunhofer IME has received funding from Dow AgroSciences for research on zinc finger nucleases.

## Supporting information


**Table S1** The efficiency, accuracy and structure of on/off‐target mutations induced by CRISPR systems in different plant species.
**Table S2** The efficiency, accuracy and structure of on/off‐target mutations induced by CRISPR systems in different animal species.
**Table S3** The efficiency, accuracy and structure of on/off‐target mutations induced by CRISPR systems in different microbial species.
**Table S4** Examples for the development of commercial plant products using different genome editing technologies and the involved IP.
