# Supplementary material for: Patterns of CRISPR/Cas9 activity in plants, animals and microbes
Source: Plant Biotechnol J. 2016 Oct 11;14(12):2203–16. doi: 10.1111/pbi.12634 (PMC5103219; doi:10.1111/pbi.12634)
Supplement: Supplementary file 1 — Table S1 The efficiency, accuracy and structure of on/off‐target mutations induced by CRISPR systems in different plant species. Table S2 The efficiency, accuracy and structure of on/off‐target mutations induced by CRISPR systems in different animal species. Table S3 The efficiency, accuracy and structure of on/off‐target mutations induced by CRISPR systems in different microbial species. Table S4 Examples for the development of commercial plant products using different genome editing technologies and the involved IP. [file PBI-14-2203-s001.docx]

**Supplementary material**

**Patterns of CRISPR/Cas9 activity in plants, animals and microbes**

Luisa Bortesi^1,+^, Changfu Zhu^2,+^, Julia Zischewski^1^, Lucia Perez^2^, Ludovic Bassié^2^, Riad Nadi^2^, Giobbe Forni^2^, Sarah Boyd Lade^2^, Erika Soto^2^, Xin Jin^2^, Vicente Medina^2^, Gemma Villorbina^2^, Pilar Muñoz^2^, Gemma Farré^2^, Rainer Fischer^1,3^, Richard M. Twyman^4^, Teresa Capell^2^, Paul Christou^2,5^ and Stefan Schillberg^3,*^

^1^Institute for Molecular Biotechnology, RWTH Aachen University, Worringerweg 1, 52074 Aachen, Germany

^2^Department of Plant Production and Forestry Science, School of Agrifood and Forestry Science and Engineering (ETSEA), University of Lleida-Agrotecnio Center, Lleida, Spain

^3^Fraunhofer Institute for Molecular Biology and Applied Ecology IME, Forckenbeckstrasse 6, 52074 Aachen, Germany

^4^TRM Ltd, PO Box 463, York YO11 9FJ, United Kingdom

^5^ICREA, Catalan Institute for Research and Advanced Studies, Passeig Lluís Companys 23, 08010 Barcelona, Spain

^+^These authors contributed equally to the work

^*^Correspondence:

Prof. Dr. Stefan Schillberg, Fraunhofer IME, Forckenbeckstrasse 6, 52074 Aachen, Germany

Tel/Fax: +49 241 6085 11050

Email: stefan.schillberg@ime.fraunhofer.de

**Supplementary Table 1.** The efficiency, accuracy and structure of on/off-target mutations induced by CRISPR systems in different plant species.

| **Species and generation/tissue^1^** | | **Input^2^** | **Repair^3^** | **Percentage efficiency and accuracy of editing^4^** | | | | | | | **Type and size of mutations^5^ (bp)** | | | | **Zygosity** | **Comments** | **Refs** |
| --- | --- | --- | --- | --- | --- | --- | --- | --- | --- | --- | --- | --- | --- | --- | --- | --- | --- |
|  |  |  |  | **CRISPR/Cas9** | | **Cas9 mod** | | | **sgRNA mod** | |  |  |  |  |  |  |  |
|  |  |  |  | **ON** | **OFF** | **ON** | **OFF** | | **ON** | **OFF** | **DEL** | **INS** | **SUB** | **COM** |  |  |  |
|  | | | | | | | | **DICOTS** | | | | | | | | | |
| Arabidopsis  (*Arabidopsis thaliana*) | P | G | NHEJ | 1.1-5.6 | NA |  |  | |  |  | 1 | 1 | 1 |  |  | Induced 7.7% small deletions (48 bp) between two sgRNAs. | Li *et al*. (2013) |
|  | L | A | NHEJ | 2.7 | NA |  |  | |  |  | 1 | 1 | 1-2 |  |  |  |  |
|  | T1 | A | NHEJ | 71.2 | NF |  |  | |  |  | 1->100 | 1-100 |  | X | 100% chimeric in T1, 22% homozygous in T2 | Predominantly 1-bp insertions and short deletions; 53% of the mutations detected in T2 plants were not found in T1 plants. | Feng *et al*. (2014) |
|  | T1 | A | NHEJ |  |  | 42.8 | NA | |  |  | 6-111 | 1-82 |  | X |  | Double nickase (52-nt overhangs) generated ~70% deletions and ~30% insertions, larger than those generated by intact Cas9. In most cases, the insertions originated directly from the sequence upstream or downstream of the insertion site. | Schiml *et al*.  (2014) |
|  | T1 | A | NHEJ | 84.7 | NF |  |  | |  |  | 1-28 | 1 | 1 |  |  | Off-target mutations not found at three sites with 3-bp mismatches using the INCURVATA 2 promoter which is active in proliferating tissues. | Hyun *et al*.  (2015) |
|  | T1 | A | NHEJ | 35.6 | NA |  |  | |  |  |  |  |  |  | 20.3% heterozygous, 8.5% chimeric, 6.8% biallelic, 1.7% biallelic | A sgRNA with 25% GC achieved a lower editing efficiency. | Ma *et al*. (2015) |
|  | T1 | A | NHEJ |  |  | 12.5 | NA | |  |  | 1-54 | 1-2 |  |  | 44% homozygous or biallelic in T2 | *St*Cas9:sgRNAs with NNAGAA-PAM: 8.6% insertions (mostly single nucleotide) and 3.8% deletions; *St*Cas9:sgRNA with NNGGAA-PAM: 11.6% insertions (primarily single nucleotide) and 3.8% deletions. | Steinert *et al*.  (2015) |
|  | T1 | A | NHEJ |  |  | 58.3-76.3 | NA | |  |  | 1-51 | 1-3 |  |  | 34% homozygous or biallelic in T2 | *Sa*Cas9: mostly insertions for NNGAA-PAM (52.1%, predominantly single nucleotide), whereas NNGGGT-PAM generated 46.7% deletions and 21.6% insertions. Both insertions and deletions were larger than those associated with NNGAA-PAM. The *Sa*Cas9 generated DSBs with at least the efficiency of *Sp*Cas9. |  |
|  | T1 | A | NHEJ | 8.3 | NF |  |  | |  |  | 1-52 | 1-16 |  | X | Up to 8.3% homozygous mutation at three loci in T1 plants | Cas9 driven by an egg-specific promoter. Off-target mutations not found at three sites with fewer than three mismatches. | Wang *et al*.  (2015b) |
|  | P | RNP | NHEJ | 16 | NF |  |  | |  |  | 1-4 | 1 |  |  |  | A 223-bp deletion between two targets. Indels were not detected at any sites that differed from the target sites by 2–5 nt. | Woo *et al*.  (2015) |
|  | T1 | A | NHEJ | 90.4 | NA |  |  | |  |  |  |  |  |  |  | Cas9 expressed in actively-dividing tissues using the YAO promoter. The CaMV 35S promoter was much less efficient (4.3%) | Yan *et al*.  (2015) |
|  | P | G | NHEJ | 6.1 | 0.5 |  |  | |  |  |  |  |  |  |  | Induced 3.7% deletions, 2.4% insertions, mostly 1-bp indels. Off-target mutations found in one of five tested sites at a frequency 13-fold lower than on-target mutations. | Sauer *et al*.  (2016) |
|  | P | G | HDR | 5.5 | NA |  |  | |  |  | 1->20 | 1 |  |  |  | Gene conversion with a 101-nt ssODN. |  |
|  | T1 | A | NHEJ | 24 | NA |  |  | |  |  |  |  |  |  |  | Combinatorial dual-sgRNA/Cas9 vector, deletions of 600–950 bp. | Zhao *et al*.  (2016) |
|  | T1 | A | HDR | 0.8 | NA |  |  | |  |  |  |  |  |  |  | Gene replacement using a combinatorial dual-sgRNA/Cas9 vector to remove 255 bp and insert a ~1.9-kb cassette with homology arms of 733 and 825 bp. |  |
| Flax  (*Linum usitatissimum*) | P | G | NEHJ | 19.8 | NF |  |  | |  |  |  |  |  |  |  | Eight potential off-target sites tested. | Sauer *et al*.  (2016) |
| Grapefruit  (*Citrus* × *paradisi*) | T0 | A | NHEJ | 100 | NF |  |  | |  |  |  | 1 |  |  | 100% chimeric | All mutants with 1-bp A/T insertions. Nine potential off-target sites tested. | Jia *et al*. (2016) |
| Lettuce  (*Lactuca sativa*) | T0 | RNP | NHEJ | 46 | NF |  |  | |  |  | 1-9 | 1 |  |  | 40% biallelic in calli | Analysis considered 91 potential off-targets that differed by 1–5 nucleotides from the on-target site. | Woo *et al*.  (2015) |
| Petunia  (*Petunia hybrida*) | P | RNP | NHEJ | 5.3-17.8 | NA |  |  | |  |  |  |  |  |  |  | Induced 63% deletions, 37% insertions. | Subburaj *et al*. (2016) |
|  | T0 | A | NHEJ | 58.1-87.5 | NA |  |  | |  |  | 1-63 | 1-2 | 1 | X | 55.6%-87.5% homozygous or biallelic in T0 | Mostly 1-bp A/T insertions. | Zhang *et al*.  (2016) |
| Poplar  (*Populus* spp) | T0 | A | NHEJ | 51.7 | NA |  |  | |  |  | 1->20 | 1 |  | X | 50% homozygous in T0 | Multiplexing with four sgRNAs, 116-bp targeted deletion and 296-bp inversion. | Fan *et al*.  (2015) |
|  | T0 | A | NHEJ | 100 | NF |  |  | |  |  | 1-50 | 1-2 |  |  | 100% biallelic in T0 | One target mostly 1-bp deletions, the other 50% deletions (mostly 2–4 bp, one 50 bp) and 50% A/T insertions (1–2 bp). | Zhou *et al*.  (2015) |
| Potato  (*Solanum tuberosum*) | T0 | A | NHEJ | 83.3 | NF |  |  | |  |  | 2-18 |  |  |  | 33.3% homozygous in T0 | Off-target analysis at one site identical to the target but without PAM. | Wang *et al*.  (2015a) |
|  | T0 | A | NHEJ | 0-29 | NA |  |  | |  |  | 2-38 | 1 |  |  |  |  | Butler *et al*. (2015) |
| Soybean  (*Glycine max*) | H | A | NHEJ | 93 | NA |  |  | |  |  | 1-54 | 1 | 1-6 | X |  | Average efficiency of 6 sgRNAs was ~54%. | Cai *et al*.  (2015) |
|  | H | A | NHEJ | 95 | 13 |  |  | |  |  | 1-8 |  | 1 | X |  | The seven most effective vectors almost exclusively induced short deletions. One off-target with two mismatches at positions 3 and 8 was modified in 2–13% of clones, compared to 95–100% at the on-target site. | Jacobs *et al*.  (2015) |
|  | E | P | NHEJ | 20 | NA |  |  | |  |  |  |  |  |  |  | Longer culture time increased editing efficiency. |  |
|  | T0 | P | HDR | 4.6 | NA |  |  | |  |  | 1->20 | 1-220 |  | X | 31.5% biallelic in calli | A 2.5-kb donor plus 1-kb homology arms on each side. | Li *et al*.  (2015b) |
|  | T0 | A | NHEJ | 48.1 | NA |  |  | |  |  | 1-38 | 1-4 |  | X |  | The sgRNA constructs were driven by the gmu6-16g-1 promoter, whereas the atu6-26 promoter achieved only 18.1% efficiency. | Du *et al*. (2016) |
| Sweet orange (*Citrus sinesis*) | L | A | NHEJ | 3.2-3.9 | NF |  |  | |  |  |  | 1-12 | 1 | X |  | *Xanthomonas citri* subsp. *citri* agroinfiltration of leaves. | Jia *et al*. (2014) |
| Tomato  (*Solanum lycopersicum*) | T0 | A | NHEJ | 48-75 | NA |  |  | |  |  | 1-26 | 1-2 |  | X | Also homozygous in T0 | Deletions of 90 bp using two sgRNAs. | Brooks *et al*. (2014) |
|  | T0 | A | NHEJ | 72.7-100 | NF |  |  | |  |  | 1->100 | 1-2 |  | X | 6.9% homozygous and 12.5% biallelic in T0 | Induced 3.3% deletions, 14.9% insertions, 11.8% combined – mostly deletions of 1–3 bp, followed by 1-bp A/T insertions. All homozygotes had 1-bp deletions, and most biallelic mutations were deletions of 1–3 bp. The three sgRNAs with a GC content >50% achieved a high editing efficiency (84–100%). No off-targets mutation at three sites for each of the four sgRNAs with 1–3 mismatches in the seed sequence. | Pan *et al*.  (2016) |
| Tobacco (*Nicotiana attenuate*) | P | RNP | NHEJ | 44 | NA |  |  | |  |  | 1 | 1 |  |  |  |  | Woo *et al*.  (2015) |
| Tobacco (*Nicotiana benthamiana*) | P | G | NHEJ | 37.7-38.5 | NA |  |  | |  |  | 1-17 | 1-216 |  | X |  |  | Li *et al*. (2013) |
|  | P | G | HDR | 9 | NA |  |  | |  |  |  |  |  |  |  | A 533-bp dsDNA donor with 114-bp homology arms, 14.2% mutation efficient by NHEJ. |  |
|  | L | A | NHEJ | 4.8 | NA |  |  | |  |  | 4-6 | 1 | 1 |  |  |  |  |
|  | L | A | NHEJ | 1.8-2.4 | NF |  |  | |  |  | 1-9 | 1 | 1 | X |  | Tested 18 off-target sites with matches of 14–17 bp to the 20-bp target sequence. | Nekrasov *et al*. (2013) |
|  | L | A/V | NHEJ | 56-30 | NA |  |  | |  |  |  |  |  |  |  | Induced mutations with an efficiency of 56% in infected leaves, 30% in systemic leaves. Agroinfiltration of transgenic plants expressing *cas9* with *Tobacco rattle* *virus* constructs carrying the sgRNA. | Ali *et al*.  (2015) |
|  | L | A/V | NHEJ | 30-50 | NA |  |  | |  |  | 1-12 | 1 | 1 | X |  | Agroinfiltration of transgenic plants expressing *cas9* with *Cabbage leaf curl virus* constructs carrying the sgRNA | Yin *et al*. (2015) |
| Tobacco (*Nicotiana tabacum*) | P | G | NHEJ | 16.2-20.3 | NA |  |  | |  |  | 1-20 | 1-2 |  | X |  | Multiplex targeted deletion and inversion of a 1.8-kb fragment. | Gao *et al*.  (2015) |
|  | T0 | A | NHEJ | 81.7-87.5 | NF |  |  | |  |  | 1-26 |  |  |  | Up to 36.4% biallelic in T0 | Off-target mutations not detected at one site with perfectly matching seed sequence. | Gao *et al*. (2015) |
|  | C | A | NHEJ | 90.5 | NA |  |  | |  |  |  |  |  |  |  | Multiplexing in BY-2 cells. Induced targeted deletions of 45, 126 and 168 bp. | Mercx *et al*. (2016) |
|  | | | | | | | | **MONOCOTS** | | | | | | | | | |
| Barley  (*Hordeum vulgare*) | T0 | A | NHEJ | 10–23 | 4.2 |  |  | |  |  | 1–3 | 1 |  | X |  | Off-target activity at sequence with a 1-bp mismatch at position 9. | Lawrenson *et al*. (2015) |
| Maize (*Zea mays*) | P | G | NHEJ | 16.4–19.1 | NA |  |  | |  |  | 1- 44 | 1-125 |  | X |  |  | Liang *et al*.  (2014) |
|  | T0 | P | NHEJ | 100 | NA |  |  | |  |  | 1-42 | 1 |  |  | 77-100 biallelic in T0 | Multiplexing with two or three sgRNAs. One plant was mutated at all six alleles. | Svitashev *et al*. (2015) |
|  | T0 | P | HDR | 0.2-0.4 | NA |  |  | |  |  |  |  |  |  |  | Induced 0.2% HDR gene modification using a plasmid with a 794-bp repair sequence as donor, or 0.3–0.4% using a 127-nt ssODN HDR template. |  |
|  | T0 | P | HDR | 2.4-4 | NA |  |  | |  |  |  |  |  |  |  | Insertion of the *pat* gene with 1-kb homology arms |  |
| Rice  (*Oryza sativa*) | T0 | A | NHEJ | 83.3-91.6 | NA |  |  | |  |  | 1-760 | 1-92 |  |  | Up to 50% homozygous in T1 |  | Miao *et al*. (2013) |
|  | P | G | NHEJ | 15-38 | ND |  |  | |  |  | 1-7 | 1 |  | X |  | No evidence of off-target mutations at one site but deletions found at another. | Shan *et al*. (2013) |
|  | T0 | P | NHEJ | 9.4 | NA |  |  | |  |  | 1–32 | 1 |  | X | 22% homozygous and 11% biallelic |  |  |
|  | T0 | A | NHEJ | 21–67 | 0-10 |  |  | |  |  | ≤ 100 | 1 | 1 | X | Up to 11% homozygous and 30.6% biallelic in T0 | No off-target mutations at 13 sites with mismatches within the seed sequence. Low-frequency mutations were found in 10% of plants at only one off-target site with a 1-bp difference outside the seed region. | Zhang *et al*.  (2014) |
|  | T0 | A | NHEJ | 20-100 | NF |  |  | |  |  | 1-43 | 1-26 |  | X | Up to 87-100% biallelic in T0 | Six potential off-target sites were analyzed. Targeted deletion of 115–245 kb in T0 plants, no detection of potential segment inversions. | Zhou *et al*. (2014) |
|  | T0 | A | NHEJ | 27.5–67.5 | 2.5–67 |  |  | |  |  | ≤ 85 | 1–2 |  |  | 5-12.5% biallelic in T0 | Accuracy differed depending on the number and position of mismatches in the sgRNA. | Li *et al*. (2016) |
|  | T0 | A | NHEJ | 42-90 | NA |  |  | |  |  | 1-34 | 1 |  | X | 14% homozygous and 76% biallelic in T0 using 1 gRNA; 48% homozygous with 2 gRNAs and 41% with 3 | Induced 42% mutations using a single sgRNA, 70% with two and 90% using three to target the same gene: ~64% deletions, ~24% insertions, and ~12% combined mutations. | Wang *et al*. (2016) |
|  | P | G | NHEJ | 3–8 | 1.6 |  |  | |  |  | 3–14 | 42–195 |  | X |  |  | Xie and Yang (2013) |
|  | T0 | A | NHEJ | 4.8–75 | NA |  |  | |  |  | 1–16 | 1 |  |  | Also biallelic in T0 |  | Feng *et al*. (2013) |
|  | T0 | A | NHEJ | 2.1– 15.6 | NF |  |  | |  |  | 1–4 | 5 | 1 | X | Also biallelic in T0 | Three highly conserved sites were identified with mismatches of 1 or 3 bp compared to the target. | Xu *et al*.  (2014) |
|  | C | A | NHEJ | 80 | X |  |  | |  |  | 1-33 | 1 | 1 |  | Also biallelic (at off-target site) | Single, double and triple mutants created with a single sgRNA by exploiting mismatch tolerance. No mutations at the 3rd, 5th and 9th off-target candidates. | Endo *et al*. (2015) |
|  | P | G | NHEJ | 42-67 | NA |  |  | |  |  |  |  |  |  |  |  | Lowder *et al*. (2015) |
|  | T0 | A | NHEJ | 33.3-53.3 | NA |  |  | |  |  | 1-9 | 1-51 |  | X | 20-40% biallelic in T0 |  |  |
|  | T0 | A | NHEJ | 84.1-90 | NA |  |  | |  |  | 1-46 | 1-2 | 1 |  | 16-39% homozygous and 51-64% biallelic in T0 |  | Ma *et al*. (2015) |
|  | T0 | A | NHEJ | 68.7 | NA |  |  | |  |  | 1-11 | 1-2 | 1 | X |  | Extension of the culture period increased the proportion of mutated cells in callus infected with *Agrobacterium tumefaciens*. | Mikami *et al*. (2015b) |
|  | C | A | NHEJ | 8.3-100 | NA |  |  | |  |  | 1-43 | 1 |  |  | Up to 80% biallelic |  | Mikami *et al*. (2015a) |
|  | T0 | A | NHEJ | 6-100 | NA |  |  | |  |  | 1-42 | 1 |  |  | Up to 76% biallelic in T0 | Multiplex expression of multiple sgRNAs from a single polycistronic vector: 45% targeted deletions of 357–761 bp in protoplasts and 6% in T0 plants. | Xie *et al*. (2015) |
|  | T0 | A | NHEJ | 85 | 0-2.2 |  |  | |  |  | 1-69 | 1-3 |  | X |  | No off-target mutations found in T0 and T1 plants without transgene, 2.2% off-target mutations in T1 plants with transgene at off-target site with 1-bp mismatch at position 13. | Xu *et al*. (2015b) |
|  | P | RNP | NHEJ | 8.4-19 | NA |  |  | |  |  | 1-5 |  |  |  |  |  | Woo *et al*. (2015) |
|  | T0 | A | HDR | 0.147-1 | NA |  |  | |  |  | 1-12 | 1-3 | 1 |  | Also biallelic gene targeting | Super-transformation of a *lig4* mutant background using ~700-bp homology region of the *ALS* gene with two nucleotide replacements: biallelic mutants recovered in T0 plants. | Endo *et al*. (2016) |
|  | T0 | P | HDR | 100 | NA |  |  | |  |  |  |  |  |  | 92% homozygous T0 | Two sgRNAs introduced with vector donor and free donor fragments (476 bp including 100 bp left and 26 bp right homology arm) and CRISPR sites flanking the donor. 75% HDR events but only heterozygous (the other allele was repaired by NHEJ). Random integration of donor was not investigated. | Sun *et al*. (2016) |
|  | C, T0 | P | NHEJ | 5 | NF |  |  | |  |  | 1–13 |  | 1 |  |  | Mutations induced in the *OsBEIIb* gene. No off-target effects at the closely-related *OsBEIIb* locus. | Baysal *et al*. (2016) |
| Sorghum (*Sorghum bicolor*) | E | A | NHEJ | 30 | NF |  |  | |  |  | 1–9 | 1 |  | X |  |  | Jiang *et al*. (2013b) |
| Wheat  (*Triticum aestivum*) | P | P | NHEJ | 28.5 | NF |  |  | |  |  | 2–10 |  |  |  |  |  | Shan *et al*. (2013) |
|  | S | A | NHEJ | 18–22 | 1–3 |  |  | |  |  | ≤ 53 | ≤ 22 |  |  |  | Duplex sgRNA/Cas9 achieved 2.8% efficiency for 53-bp deletions between sites. | Upadhyay *et al*. (2013) |

Footnotes:

^1^ – T0 = T0 plants, T1 = T1 plants, P = protoplasts, C = callus, E = embryos, L = leaves (transient expression), S = cell suspension culture, H = hairy roots.

^2^ – A = Agrobacterium, A/V = Agrodelivery of viral DNA, G = PEG transformation, P = particle bombardment, RNP = delivery of Cas9-sgRNA ribonucleoprotein complex.

^3^ – HDR = homology-dependent repair, NHEJ = non-homologous end joining.

^4^ – CRISPR/Cas9 = standard CRISPR/Cas9 system, Cas mod = some form of modified Cas9 (mutation), sgRNA mod = some form of modified gRNA (e.g. truncated, extended, double target). In these columns, efficiency and accuracy are defined by the percentage of on-target and off-target mutations. Efficiency = (on-target + off-target)/total attempts. Accuracy = on-target/total attempts. NF = analysis was carried out by the authors but no mutations of this category were found. ND = analysis was carried out but the frequency was not determined.

^5^ – DEL = deletion, INS = insertion, SUB = substitution, COM = combination (an X in this column shows that combinations of DEL, INS and/or SUB events were recovered).

**Supplementary Table 2.** The efficiency, accuracy and structure of on/off-target mutations induced by CRISPR systems in different animal species.

| **Species and tissue^1^** | | **Input^2^** | **Repair^3^** | **Percentage efficiency and accuracy of editing^4^** | | | | | | **Type and size of mutations^5^ (bp)** | | | | **Comments** | **Refs** |
| --- | --- | --- | --- | --- | --- | --- | --- | --- | --- | --- | --- | --- | --- | --- | --- |
|  |  |  |  | **CRISPR/Cas9** | | **Cas9 mod** | | **sgRNA mod** | |  |  |  |  |  |  |
|  |  |  |  | **ON** | **OFF** | **ON** | **OFF** | **ON** | **OFF** | **DEL** | **INS** | **SUB** | **COM** |  |  |
| **INVERTEBRATES** | | | | | | | | | | | | | | | |
| Nematode (*Caenorhabditis elegans*) | G | M | NHEJ | 0.5-80.3 | NA |  |  |  |  | 1-13 | 1-15 |  | X |  | Friedland *et al*. (2013) |
|  | G | M | HDR | 3.33 | NA |  |  |  |  |  |  |  |  | Off-target analysis with 6-9 mismatches. | Dickinson *et al*. (2013) |
| Fruit fly (*Drosophila melanogaster*) | E | M | NHEJ | 4-88 | NA |  |  |  |  |  |  |  |  | Induced 4–88% mutations in F0 generation, 0.25–98.9% in F1. | Bassett *et al*. (2014) |
| Mosquito (*Culex quinquefasciatus*) | E | M | NHEJ | ~3.1 | NA |  |  |  |  |  |  |  |  | Efficiency of ~3% in F1 males (germline mutation). | Itokawa *et al*. (2016) |
| **VERTEBRATES, NON-MAMMALS** | | | | | | | | | | | | | | | |
| Zebrafish  (*Danio rerio*) | E | M | NHEJ, HDR | 24-59, | NA |  |  |  |  |  |  |  |  | Induced 42–100% germline transmission. | Auer and Del Bene (2014) |
|  | E | M | NHEJ | 24.1-59.4 | NA |  |  |  |  |  |  |  |  | Success with 80% of sgRNAs. | Hwang *et al*. (2013) |
|  | E | M | NHEJ, HDR | 86 NHEJ, 3.5-15.6 HDR | 1.1-2.5 |  |  |  |  |  |  |  |  |  | Hruscha *et al*. (2013) |
| Chicken  (*Gallus gallus*) | PGC | T | NHEJ | >90 | NF |  |  |  |  |  |  |  |  |  | Oishi *et al*. (2016) |
| **VERTEBRATES, NON-HUMAN MAMMALS** | | | | | | | | | | | | | | | |
| Mouse  (*Mus musculus*) | ST | M | NHEJ | 3.7-80.1 | 0-22 |  |  |  |  | 5-31 |  |  |  | Mutations frequency of 3.7-80.1%. Mutations at sites 1–3: 23.6%, 30.1% and 10.9% in cells; 3.7%, 35.8% and 27.8% in animals. Compared standard (20-nt) and truncated (17-18 nt) sgRNAs, the latter performing better. One off-target mutation (29% with truncated sgRNA, 3.7% with standard sgRNA). | An *et al*. (2016) |
|  | E | M | NHEJ | 3.7- 35.8 | NF |  |  |  |  | 5-31 |  |  |  |  |  |
|  | E, ST | M | NHEJ | >80 | NA |  |  |  |  |  |  |  |  |  | Yang *et al*. (2014) |
|  | E, ST | M | HDR | 10-80 | NA |  |  |  |  |  |  |  |  | Induced 10–80% mutation efficiency with ssODN, 10–30% with plasmid. |  |
|  | ST | T | NHEJ | 12-37 | 0.7 |  |  |  |  |  |  |  |  | Only one among 295 off-target candidate sites identified by dCas9 binding was mutated. | Wu *et al*. (2014) |
|  | ST | E | NHEJ | 30-44 | NA |  |  |  |  |  |  |  |  |  | Platt *et al*, (2014) |
|  | ST | E | HDR | 4 | NA |  |  |  |  |  |  |  |  |  |  |
|  | ST | T | NHEJ | 12.5-100 | NF |  |  |  |  | 1-251 |  |  |  | Multiplex targeting of six genes, 3–5 mutated per clone, majority of deletions 1-40 bp. | Heckl *et al*. (2014) |
|  | LC | V | NHEJ | 95 | NF |  |  |  |  | 3 -23 | 1 |  | X | Adenoviral delivery. | Cheng *et al*. (2014) |
|  | LT | V | NHEJ | 90 | NF |  |  |  |  | 39-56 | 67 |  |  |  |  |
|  | ST, E | M | NHEJ, HDR | 36-48 | NA |  |  |  |  |  |  |  |  | Mutation frequencies of 48% for single loci, 21% at three loci, 10% at five loci. Three targets separately: 36%, 36% and 48%. Multiplexed three genes: 21.8% indels in six alleles of three genes. Multiplexed five genes: 10% indels in all eight alleles of five genes. HDR in 10 pups, seven at locus 1, eight at locus 2, and six at both. | Wang *et al*. (2013) |
| Rabbit (*Oryctolagus cuniculus*) | E | M | NHEJ | 80 | NF |  |  |  |  |  |  |  |  |  | Lv *et al*. (2016) |
| Rat  (*Rattus norvegicus*) | ST | T | NHEJ | 10 | NA |  |  |  |  |  |  |  |  | Mutation frequency of ~10%, approximately half of which were frameshifts. | Chapman *et al*. (2015) |
|  | E | M | NHEJ |  |  | 39-72 | NA |  |  |  |  |  |  | Cas9 nickase plus two sgRNAs. F0 generation: 72% locus 1, 39% locus 2, 28% mutated at both loci. HDR with 700-bp arms flanking 3-kb insert in 6% of F0 pups. | Shao *et al*. (2014) |
|  | E | M | HDR |  |  | 17 | NA |  |  |  |  |  |  |  |  |
| Sheep  (*Ovis aries*) | E | M | HDR | 12.5 | NA |  |  |  |  |  |  |  |  | Mutation frequency of 12.5% using homology arms of 1 kb each, plasmid delivery. | Wu *et al*. (2016) |
| **HUMAN CELLS** | | | | | | | | | | | | | | | |
| Human  (*Homo sapiens*) | ST | T | NHEJ | 12-37 | 0.7 |  |  |  |  |  |  |  |  | Only one among 295 off-target candidate sites identified by dCas9 binding was mutated. | Wu *et al*. (2014) |
|  | ST | E | HDR | 0.3-8.3 | NF |  |  |  |  |  |  |  |  | Incorporation of CRISPR-blocking mutations in PAM or sgRNA achieved a 2–10-fold increase in HDR accuracy. | Paquet *et al*. (2016) |
|  | ST | T | NHEJ, HDR | 0-8 | NA | Cas9n, dCas9-FokI |  |  |  |  |  |  |  | Comparison of three cell types and different nucleases: overall very low efficiencies. | Miyaoka *et al*. (2016) |
|  | ST | T | HDR | 2.8 | NF |  |  |  |  |  |  |  |  |  | Zhu *et al*. (2015) |
|  | ST | E | NHEJ | 88 |  |  |  |  |  | 52% | 32% | 4% |  | Comparison of 11 cell types, transfection/electroporation and delivery of plasmid, mRNA or RNP (RNP plus electroporation performed best for all 11 cell types). | Liang *et al*. (2015) |
|  | ST, C, EC | E | NHEJ | 94 | See comment |  |  |  |  | 16% | 75% | 5% |  | Jurkat cells: 1.6–28-fold lower ratio of off-target mutations at a known off-target for RNP-transfected cells compared to DNA-transfected cells. Multiplex approach achieved frequencies of 93% (two loci) and 65% (three loci). |  |
|  | C | T | NHEJ | 88 | 0.11-85 |  |  | GGx sgRNA: 4.8-37.8 | GGx: 0.01-1.3 | 1-19  1-42 | 1  1 |  | X | HAP1 cells: 5'GG in sgRNA reduces off-target activity, but (depending on the sgRNA) can also reduce on-target activity. Off-target activity observd for promiscuous sgRNAs. | Kim *et al*. (2015) |
|  | EC, C | T | NHEJ | 33-91.5 | NA |  |  | Tru-sgRNA |  |  |  |  |  | Efficiency of 33–36% and 67.4–91.5%. An sgRNAs shorter than 16 nt showed almost no indel formation at target site, but transcriptional activation with Cas9 (up to 10,000-fold). Truncated sgRNAs showed similar mutation frequencies to standard sgRNAs. | Dahlman *et al*. (2015) |
|  | EC, C |  | HDR | 50-66 | NA |  |  |  |  |  |  |  |  | HEK cells: suppression of key NHEJ molecules by gene silencing, ligase inhibitor or coexpression of *Adenovirus* protein increased HDR efficiency by up to 8-fold. | Chu *et al*. (2015) |
|  | ST | E | NHEJ | 57-76 | 2-5 mutations per clone |  |  |  |  |  |  |  |  | Whole genome sequencing showed that each individual clone had 2–5 off-target events caused by the nuclease, but also an average of 100 unique single nucleotide variants (likely to have spontaneously arisen in culture). | Veres *et al*. (2014) |
|  | ST | T | HDR | NA | NF |  |  |  |  |  |  |  |  | Whole genome sequencing showed that none of the identified single nucleotide variants and indels directly resulted from nuclease activity. | Smith *et al*. (2014) |
|  | EC | T | NHEJ | 1.6-8 | NA |  |  |  |  |  |  |  |  | HEK cells: efficiency reflects structural rearrangements (chromosomal). | Choi and Meyerson (2014) |
|  | ST | T | NHEJ, HDR | 1.2 NHEJ, 1.7 HR | NA |  |  |  |  |  |  |  |  | Observed moderate but statistically significant correlation between NHEJ efficiency and melting temperature of the sgRNA spacer sequence. | Yang *et al*. (2013) |
|  | EC, C | T | NHEJ | 3.33-50.2 | 0-150 |  |  |  |  |  |  |  |  | U2OS, HEK and K562 cells. Analysis of off-target sites containing 1–5 mismatches. | Fu *et al*. (2013) |

Footnotes:

^1^ – C = Cancer-derived cell line, E = embryos, EC = embryonic cell line, G = gonad, LC = liver cells, LT = liver tissue, PGC = primordial germ cells, ST = stem cells.

^2^ – M = microinjection, T = transfection, E = electroporation, V = viral delivery.

^3^ – HDR = homology-dependent repair, NHEJ = non-homologous end joining.

^4^ – CRISPR/Cas9 = standard CRISPR/Cas9 system, Cas mod = some form of modified Cas9 (e.g. mutation), sgRNA mod = some form of modified gRNA (e.g. truncated, extended, double target). In these columns, efficiency and accuracy are defined by the percentage of on-target and off-target mutations. Efficiency = (on-target + off-target)/total attempts. Accuracy = on-target/total attempts. NF = analysis was carried out by the authors but no mutations of this category were found. ND = analysis was carried out but the frequency was not determined.

^5^ – DEL = deletion, INS = insertion, SUB = substitution, COM = combination (an X in this column shows that combinations of DEL, INS and/or SUB events were recovered).

**Supplementary Table 3.** The efficiency, accuracy and structure of on/off-target mutations induced by CRISPR systems in different microbial species.

| **Species** | **Input^1^** | **Repair^2^** | **Percentage efficiency and accuracy of editing^3^** | | | | | | **Type and size of mutations^4^ (bp)** | | | | **Comments** | **Refs** |
| --- | --- | --- | --- | --- | --- | --- | --- | --- | --- | --- | --- | --- | --- | --- |
|  |  |  | **CRISPR/Cas9** | | **Cas9 mod** | | **sgRNA mod** | |  |  |  |  |  |  |
|  |  |  | **ON** | **OFF** | **ON** | **OFF** | **ON** | **OFF** | **DEL** | **INS** | **SUB** | **COM** |  |  |
| **EUKARYOTES** | | | | | | | | | | | | | | |
| *Chlamydomonas reinhardtii* | E | NHEJ | NA | NA |  |  |  |  | 1-2 |  | 1-9 |  | Only transient experiments, no frequency determined. Failure to recover transformants with intact or expressed *cas9* genes following transformation with the *cas9* gene alone. | Jiang *et al*. (2014) |
| *Phaeodactylum tricornutum* | P | NHEJ | 25-63 | NA |  |  |  |  | 1-137 | 1-212 | 1 | X | The 212-bp insertion consisted of fragments of the vectors used for transformation. | Nymark *et al*. (2016) |
| *Pichia pastoris* | E | NHEJ | 94 | NA |  |  |  |  |  |  |  |  |  | Weninger *et al*. (2016) |
| *Saccharomyces cerevisiae* | T | HDR | ~100 | NA |  |  |  |  |  |  |  |  | Co-transformation of a sgRNA plasmid and donor DNA in cells constitutively expressing *cas9*. | DiCarlo *et al*. (2013) |
|  | T | HDR | 65-78 | NA |  |  |  |  |  |  |  |  | Diploid industrial strains: simultaneous disruption of two endogenous genes and insertion of a heterologous gene, with 40-bp homology sequences. | Stovicek *et al*. (2015) |
|  | T | HDR | 27-87 | NA |  |  |  |  |  |  |  |  | Disruption of three genes simultaneously by multiplexing. A 100-bp donor sequence was designed with two 50-bp homology arms flanking the Cas9 cutting site. | Bao *et al*. (2015) |
|  | T | HDR | 25-100 | NA |  |  |  |  |  |  |  |  | Genes deleted and three inserted simultaneously. | Tsai *et al*. (2015a) |
|  | T | HDR | 30-100 | NA |  |  |  |  |  |  |  |  | Seamless introduction of single nucleotide changes at any location using a two-step procedure. | Biot-Pelletier *et al*. (2016) |
| *Plasmodium falciparum* | E | HDR | 100 | NF |  |  |  |  |  |  |  |  | No evidence of off-target effects by whole genome sequencing. | Ghorbal *et al*. (2014) |
|  | E | HDR | >50-100 | NA |  |  |  |  |  |  |  |  |  | Wagner *et al*. (2014) |
| **BACTERIA** | | | | | | | | | | | | | | |
| *Streptococcus pneumoniae* | T | HDR | 99 | NA |  |  |  |  |  |  |  |  | Multiplexing by ssODN-mediated HDR. The approach relies on dual-sgRNA:Cas9-directed cleavage at the target site to kill wild-type cells and circumvents the need for selectable markers or counterselection systems. | Jiang *et al*. (2013a) |
| *Escherichia coli* | E | HDR | 65 | NA |  |  |  |  |  |  |  |  |  |  |
| *Lactobacillus reuteri* | E | HDE | 90-100 | NA |  |  |  |  |  |  |  |  | Strategy was ssDNA-mediated recombineering. | Oh and van Pijkeren (2014) |
| *Clostridium beijerinckii* | E | HDR | 100 | NA |  |  |  |  |  |  |  |  | Deletion of 262 bp using a donor with 1-kb homology arms. DNA editing template onto the same vector with the cas9 and sgRNA genes. Approximately 50% if of the colonies carried only the desired deletion and no integrated plasmid. | Wang *et al*. (2015c) |
| *Clostridium cellulolyticum* | E | HDR |  |  | >95 | NA |  |  |  |  |  |  | Cas9 nickase, 200-bp homolog arms. | Xu *et al*. (2015c) |
| *Clostridium pasteurianum* | E | HDR | 100 | NA |  |  |  |  |  |  |  |  | Frequency of 25% edited cells compared with the endogenous Type IB CRISPR/Cas system. Deletion of 567 bp using ~1-kb homology arms. Expression of *cas9* in the absence of a sgRNA significantly reduces transformation efficiency. | Pyne *et al*. (2015) |

Footnotes:

^1^ – E = electroporation, P = particle bombardment, T = transfection.

^2^ – HDR = homology-dependent repair, NHEJ = non-homologous end joining

^3^ – CRISPR/Cas9 = standard CRISPR/Cas9 system, Cas mod = some form of modified Cas9 (e.g. mutation), sgRNA mod = some form of modified gRNA (e.g. truncated, extended, double target). In these columns, efficiency and accuracy are defined by the percentage of on-target and off-target mutations. Efficiency = (on-target + off-target)/total attempts. Accuracy = on-target/total attempts. NF = analysis was carried out by the authors but no mutations of this category were found. ND = analysis was carried out but the frequency was not determined.

^5^ – DEL = deletion, INS = insertion, SUB = substitution, COM = combination (an X in this column shows that combinations of DEL, INS and/or SUB events were recovered).

**Supplementary Table 4.** Examples for the development of commercial plant products using different genome editing technologies and the involved IP.

| **Company** | **IP situation^1^** | **Plant^1^** | **Trait^1^** | **Development phase^1^** | **Reference** |
| --- | --- | --- | --- | --- | --- |
| ODM | | | | | |
| Cibus^TM^ | Own IP | Canola | Sulfonylurea herbicide-tolerant | Launched in the US in 2015, expected in Canada in 2017 and in other major global markets in 2018 | www.cibus.com/products.php (accessed 3 June 2016) |
|  |  | Flax | Glyphosate-tolerant | Expected launch in the US in 2019, and in Canada in 2020 |  |
|  |  | Potato | *Phytophthora infestans*-resistant | Launch expected first in the US in late 2019 |  |
|  |  | Rice | Herbicide-tolerant | Launch expected first in the US |  |
| ZFN | | | | | |
| Dow AgroSciences | License from Sangamo BioSciences | Maize | Sequential integration of two herbicide resistance genes into the same genomic loci | Development phase | Ainley *et al*. (2013) |
| TALEN | | | | | |
| Calyxt^2^ | Licenses from University of Minnesota and Iowa State University Research Foundation, cross-license agreement with Two Blades Foundation | Canola | Oil with lower levels of saturated fat | Development phase | http://www.calyxt.com/products/lower-saturated-fat-canola-oil/ (assessed 3 June 2016) |
|  |  | Potato | Cold storable | First field trial completed in 2015 | Clasen *et al*. (2016), http://www.calyxt.com/wp-content/uploads/2015/11/PR_Calyxt_Potato_Field_Trial.pdf (assessed 3 June 2016 |
|  |  | Soybean | High oleic acid and low linoleic acid content | Production of 30 tons in Argentina, launch expected in 2018 | Haun *et al*. (2014), http://www.calyxt.com/wp-content/uploads/2016/05/PR-5.24.16_Calyxt_Argentina_Soybean.pdf (assessed 3 June 2016) |
|  |  | Wheat | Reduced gluten | Development phase | http://www.calyxt.com/products/gluten-reduced-wheat/ (assessed 3 June 2016) |
| CRISPR | | | | | |
| DuPont Pioneer | Own IP and licenses from Vilnius University and Caribou Biosciences | Maize | Drought-resistant | Launch expected in 5–10 years | https://www.technologyreview.com/s/542311/dupont-predicts-crispr-plants-on-dinner-plates-in-five-years/ (assessed 3 June 2016) |
|  |  | Maize | Waxy hybrids with improved starch composition | Launch expected within 5 years | https://www.plantmanagementnetwork.org/pub/crop/news /2016/CRISPRCas/ (assessed 3 June 2016) |

Footnotes:

^1^ Based on publicly available information.

^2^ Formerly known as Cellectis.

**Supplementary references** (references in supplementary tables that are not also cited in the main text)

Ainley, W.M., Sastry-Dent, L., Welter, M.E., Murray, M.G., Zeitler, B., Amora, R., Corbin, D.R., Miles, R.R., Arnold, N.L., Strange, T.L., Simpson, M.A., Cao, Z., Carroll, C., Pawelczak, K.S., Blue, R., West, K., Rowland, L.M., Perkins, D., Samuel, P., Dewes, C.M., Shen, L., Sriram, S., Evans, S.L., Rebar, E.J., Zhang, L., Gregory, P.D., Urnov, F.D., Webb, S.R. and Petolino, J.F. (2013) Trait stacking via targeted genome editing. *Plant Biotechnol. J.* **11**, 1126­–1134.

Ali, Z., Abul-Faraj, A., Li, L., Ghosh, N., Piatek, M., Mahjoub, A., Aouida, M., Piatek, A., Baltes, D.F., Dinesh-Kumar, S. and Mahfouz, M.M. (2015) Virus-mediated genome editing in plants using the CRISPR/Cas9 system. *Mol. Plant*, **8**, 1288–1291.

Auer, T.O. and Del Bene, F. (2014) CRISPR/Cas9 and TALEN-mediated knock-in approaches in zebrafish. *Methods*, **69**, 142–150.

Butler, N.M., Atkins, P.A., Voytas, D.F. and Douches, D.S. (2015) Generation and inheritance of targeted mutations in potato (Solanum tuberosum L.) using the CRISPR/Cas system. *PLOS One*, **10**, e0144591.

Chapman,K.M., Medrano, G.A., Jaichander, P., Chaudhary, J., Waits, A.E., Nobrega, M.A., Hotaling, J.M., Ober, C. and Hamra, F.K. (2015) Targeted germline modifications in rats using CRISPR/Cas9 and spermatogonial stem cells. *Cell Rep.* **10**, 1828–1835.

Clasen, B.M., Stoddard, T.J., Luo, S., Demorest, Z.L., Li, J., Cedrone, F., Tibebu, R., Davison, S., Ray, E.E., Daulhac, A., Coffman, A., Yabandith, A., Retterath, A., Haun, W., Baltes, N.J., Mathis, L., Voytas, D.F. and Zhang, F. (2016) Improving cold storage and processing traits in potato through targeted gene knockout. *Plant Biotechnol. J.* **14**, 169–176.

Dahlman, J.E, Abudayyeh, O.O., Joung, J., Gootenberg, J.S., Zhang, F. and Konermann, S. (2015) Orthogonal gene knockout and activation with a catalytically active Cas9 nuclease. *Nature Biotechnol.* **33**, 1159–1161.

Du, H., Zeng, X., Zhao, M., Cui, X., Wang, Q., Yang, H., Cheng, H. and Yu, D. (2016) Efficient targeted mutagenesis in soybean by TALENs and CRISPR/Cas9. *J. Biotechnol*. **217**, 90–97.

Feng, Z., Zhang, B., Ding, W., Liu, X., Yang, D.L., Wei, P., Cao, F., Zhu, S., Zhang, F., Mao, Y. and Zhu, J.K. (2013) Efficient genome editing in plants using a CRISPR/Cas system. *Cell Res*. **23**, 1229–1232.

Haun, W., Coffman, A., Clasen, B.M., Demorest, Z.L., Lowy, A., Ray, E., Retterath, A., Stoddard, T., Juillerat, A., Cedrone, F., Mathis, L., Voytas, D.F. and Zhang, F. (2014) Improved soybean oil quality by targeted mutagenesis of the fatty acid desaturase 2 gene family. *Plant Biotechnol. J.* **12**, 934–940.

Itokawa, K., Komagata, O., Kasai, S., Ogawa, K. and Tomita, T. (2016) Testing the causality between CYP9M10 and pyrethroid resistance using the TALEN and CRISPR/Cas9 technologies. *Sci. Rep.* **6**, 24652.

Jia, H. and Wang, N. (2014) Targeted genome editing of sweet orange using Cas9/sgRNA. *PLOS One*, **9**, e93806.

Li, Z., Liu, Z.B., Xing, A., Moon, B.P., Koellhoffer, J.P., Huang, L., Ward, R.T., Clifton, E., Falco, S.C. and Cigan, A.M. (2015b) Cas9-guid RNA directed genome editing in soybean. *Plant Physiol.* **169**, 960–970.

Liang, Z., Zhang, K., Chen, K. and Gao, C. (2014) Targeted mutagenesis in Zea mays using TALENs and the CRISPR/Cas system. *J. Genet. Genom*. **41**, 63–68.

Mercx, S., Tollet, J., Magy, B., Navarre, C. and Boutry, M. (2016) Gene inactivation by CRISPR-Cas9 in Nicotiana tabacum BY-2 suspension cells. *Front. Plant Sci*. **7**, 40.

Nekrasov, V., Staskawicz, B., Weigel, D., Jones, J.D. and Kamoun, S. (2013) Targeted mutagenesis in the model plant Nicotiana benthamiana using Cas9 RNA-guided endonuclease. *Nature Biotechnol.* **31**, 691–693.

Oh, J-H. and van Pijkeren, J-P. (2014) CRISPR–Cas9-assisted recombineering in Lactobacillus reuteri. *Nucleic Acids Res*. **42**, e131.

Paquet, D., Kwart, D., Chen, A., Sproul, A., Jacob, S., Teo, S., Olsen, K.M., Gregg, A., Noggle, S. and Tessier-Lavigne, M. (2016) Efficient introduction of specific homozygous and heterozygous mutations using CRISPR/Cas9. *Nature*, **533**, 125–129.

Pyne, M.E., Bruder, M.R., Moo-Young, M., Chung, D.A. and Chou, C.P. (2016) Harnessing heterologous and endogenous CRISPR-Cas machineries for efficient markerless genome editing in Clostridium. *Sci. Rep*. **6**, 25666.

Wagner, J.C., Platt, R.J., Goldfless, S.J., Zhang, F. and Niles, J.C. (2014) Efficient CRISPR-Cas9-mediated genome editing in Plasmodium falciparum. *Nature Methods*, **11**, 915–918.

Wang, Y., Zhang, Z-T., Seo, S-O., Choi, K., Lu, T., Jin, Y-S., Blaschek, H.P. (2015c) Markerless chromosomal gene deletion in Clostridium beijerinckii using CRISPR/Cas9 system. *J. Biotechnol*. **200**, 1–5.

Wu, X., Scott, D.A., Kriz, A.J., Chiu, A.C., Hsu, P.D., Dadon, D.B., Cheng, A.W., Trevino, A.E., Konermann, S., Chen, S., Jaenisch, R., Zhang, F. and Sharp, P.A. (2014) Genome-wide binding of the CRISPR endonuclease Cas9 in mammalian cells. *Nature Biotechnol.* **32**, 670–676.

Wu, M., Wei, C., Lian, Z., Liu, R., Zhu, C., Wang, H., Cao, J., Shen, Y., Zhao, F., Zhang, L., Mu, Z., Wang, Y., Wang, X., Du, L. and Wang, C. (2016) Rosa26-targeted sheep gene knock-in via CRISPR-Cas9 system. *Sci. Rep.* **6**, 24360.

Xu, R., Li, H., Qin, R., Wang, L., Li, L., Wei, P. and Yang, J. (2014) Gene targeting using the Agrobacterium tumefaciens-mediated CRISPR-Cas system in rice. *Rice*, **7**, 5.

Yin, K., Han, T., Liu, G., Chen, T., Wang, Y., Yu, A.Y.L. and Liu, Y. (2015) A geminivirus-based guide RNA delivery system for CRISPR/Cas9 mediated plant genome editing. *Sci. Rep*. **5**, 14926.
